# Secondary Metabolites from the Cultures of Medicinal Mushroom *Vanderbylia robiniophila* and Their Tyrosinase Inhibitory Activities

**DOI:** 10.3390/jof9070702

**Published:** 2023-06-26

**Authors:** Yuxi Wang, Jinghui Jia, Qi Wang, Yulian Wei, Haisheng Yuan

**Affiliations:** 1 CAS Key Laboratory of Forest Ecology and Management, Institute of Applied Ecology, Chinese Academy of Sciences, Shenyang 110164, China; wangyuxi@iae.ac.cn (Y.W.); jjh1821129@163.com (J.J.); wqi202306@163.com (Q.W.); weiyulian@iae.ac.cn (Y.W.); 2 College of Life Sciences, Liaoning University, Shenyang 110036, China; 3 School of Life Sciences and Biopharmaceutics, Shenyang Pharmaceutical University, Shenyang 110016, China

**Keywords:** *Vanderbylia robiniophila* (Huaier), secondary metabolites, tyrosinase inhibitory activity, inhibition mechanism

## Abstract

*Vanderbylia robiniophila* (Huaier in Chinese) has been used as a traditional herbal medicine in China for over 1600 years. However, the secondary metabolites of *V. robiniophila* have not been systematically examined. Corresponding chemical investigation in this study led to the discovery of two new compounds, (22*E*, 24*R*)-6*β*, 7*α*-dimethoxyergosta-8(14), 22-diene-3*β*, 5*α*-diol (**1**) and vanderbyliolide A (**8**), along with eight known ones (**2**–**7**, **9**–**10**). Their structures were determined by extensive spectroscopic analyses and electronic circular dichroism (ECD) calculations. The tyrosinase inhibitory activity of all isolated compounds was evaluated, and compound **10** showed a potential tyrosinase inhibitory effect with an IC_50_ value of 60.47 ± 2.63 μM. Kinetic studies of the inhibition reactions suggested that **10** provides the inhibitory ability on tyrosinase in an uncompetitive way.

## 1. Introduction

Melanin is the primary pigment responsible for skin color and also protects human skin against harmful effects by absorbing ultraviolet (UV) rays and mitigating oxidative stress [1]. The excessive production of melanin can lead to hyperpigmentation-related disorders and even melanoma in severe cases [2]. Tyrosinase is a reaction rate-limiting enzyme in the process of melanogenesis [3,4]. The skin-lightening products have been investigated as the melanogenesis inhibitors in the medical and cosmetic industry. They are in large demand with a worldwide market of USD 23 billion in 2020 [5]. However, most of the commercially available tyrosinase inhibitors have some drawbacks. For instance, vitamin C is susceptibly degradable and sensitive to temperature and air [6]. Kojic acid and arbutin are reported to cause safety issues such as skin irritation [7], while arbutin is chemically instable and might result in leukemia due to the metabolized products of benzene analogues [8]. Therefore, safe, stable, and effective tyrosinase inhibitors are still in great need for the treatment of dyspigmentation disease and cosmetic applications.

Mushrooms have been traditionally used as food ingredients and folk medicine since antiquity, and recent studies have elucidated their functional benefits, such as anticancer, anti-inflammatory, antiviral, antibacterial and hepatoprotective properties, which have been attributed to various mushroom components [9,10]. Medicinal mushrooms are known for producing specialized molecules with great chemo-diversity that have relevant impacts on human health and diseases [11]. These compounds have proved immensely valuable in the pharmaceutical industry as they provide the structural template and pharmacophores of commercially successful drugs with excellent clinical efficacies and acceptable side-effects [12]. These include the antibiotic retapamulin from *Pleurotus multilus* [13] and the first-in-class sphingosine-1-phosphate (S1P) receptor modulator named fingolimod from *Isaria sinclairii* [14]. *Vanderbylia robiniophila* (Murrill) B.K. Cui & Y.C. Dai (Huaier) is a medicinal mushroom mainly parasitized on the trunk of *Robinia pseudoacacia* L. It has been widely used for more than 1600 years in traditional Chinese medicine (TCM) [15]. Nowadays, “Huaier granule” is recognized by Chinese State Food and Drug Administration as a complementary medicine for the treatment of multiple malignancies, including liver cancer, lung cancer, digestive system cancers, and breast carcinoma [16,17].

The mechanisms behind the significant antitumor effect exerted by *Vanderbylia robiniophila* are fascinating and intrigue many researchers [18]. It has been revealed that *V. robiniophila* directly inhibits the growth and proliferation of cancer cells [19], arrests the cell cycle [20], restrains invasion and metastasis [21], interferes with angiogenesis [22], induces cell apoptosis [23] and regulates immune responses [24]. In contrast to the pharmacological activities, there is limited information about the chemical constituents of *V. robiniophila*. Recent studies have shown that polysaccharides, proteoglycan, and amino acids are the primary ingredients in *V. robiniophila* extract [25,26]. A metabolomic comparison between the naturally and the artificially cultured Huaier extract using LC-MS analysis showed that the former contains more amino acids, alkaloids, and terpenoids [27]. However, few researchers have been able to conduct any systematic research into the isolation and exact structure elucidation of the secondary metabolites of *V. robiniophila* [28].

**Figure 1:**
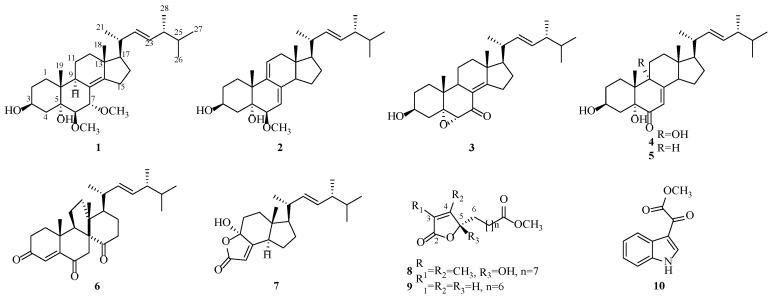
Chemical structures of compounds **1**–**10**.

During our investigations on macrofungal resources, a wild-derived strain was isolated from the fruit body of *Vanderbylia robiniophila*, and the EtOAc fraction of the strain showed significant tyrosinase inhibitory activity. To discover potential tyrosinase inhibitors in *V. robiniophila*, a detailed chemical investigation of the large-scale fermentation in rice was carried out, which resulted in the purification of two novel secondary metabolites, along with eight known ones, including seven steroids (**1**–**7**), two 2(5*H*)-furanone derivatives (**8**–**9**), and a monoindole alkaloid (**10**) (Figure 1). The tyrosinase inhibitory activities of the isolated compounds were evaluated. Herein, the details of the isolation, structure elucidation, and bioactivities of compounds **1**–**10** are described.

## 2. Materials and Methods

### 2.1. General Experimental Procedures

UV spectra were recorded on an ultraviolet-visible spectrometer (UV-1500PC, Macy Instruments, Inc., Shanghai, China) and IR spectra were measured on a Thermo Fisher Nicolet 6700 FT-IR spectrometer (Thermo Scientific, Madison, WI, USA). Optical rotation values were obtained using a JASCO DIP-370 digital polarimeter (JASCO, Tokyo, Japan) at 20 °C. The ECD spectrum was measured by a BioLogic Science MOS-450 spectrometer (Bio-Logic Science Instruments, Seyssinet-Pariset, France). HRESIMS data were obtained in the positive-ion mode on a Waters Xevo G2-S QTOF mass spectrometer (Waters Corporation, Milford, MA, USA). 1D and 2D NMR were tested using a Bruker AVANCE NEO 600 MHz NMR spectrometer (Bruker Corporation, Bremen, Germany), where chemical shifts (*δ*) are given in parts per million (ppm) units with tetramethylsilane (TMS) as an internal standard. Chromatographic silica gel (100–200 and 200–300 mesh, Qingdao Marine Chemical Factory, Qingdao, China), DIAION HP-20 macroporous adsorption resin (250–850 μm, Mitsubishi Chemical Corporation, Tokyo, Japan), and reversed-phase C18 (RP-C18) silica gel (50 μm, YMC Co., Ltd., Kyoto, Japan) were used for column chromatography. Thin-layer chromatography (TLC) (GF_254_, 10–40 μm, Qingdao Bangkai Hi-Tech Materials Co., Ltd., Qingdao, China) was employed to monitor the fractions. TLC spots were visualized by UV light (254 and 365 nm) and further reacted with 20% sulfuric acid spray reagent solution under the condition of heat. HPLC was conducted with an LC-20AT liquid chromatography system equipped with a Shimadzu SPD-20A UV/VIS detector and a Shimadzu RID-20A RI detector (Shimadzu, Kyoto, Japan) using a YMC Pack ODS-A column (250 × 10 mm, 5 μm, YMC Company, Kyoto, Japan). The OD value was determined using a BioTek PowerWave XS2 microplate reader (BioTek, Winooski, VT, USA).

### 2.2. Reagents and Chemicals

The HPLC-grade solvents, such as methanol and acetonitrile, and the analytical reagent solvents, such as petroleum ether, dichloromethane, ethyl acetate, and ethanol, were purchased from Tianjin Yongda Chemical Reagent Co., Ltd. (Tianjin, China). Tyrosinase (1100 U/mg) from Shanghai Yuanye Biotechnology Co., Ltd. (Shanghai, China) was used as the enzyme. L-tyrosine (98%, Biotopped, Beijing, China) served as the substrate and arbutin (98%, Shanghai Macklin Biochemical Co., Ltd., Shanghai, China) was used as a positive control.

### 2.3. Fungal Material

*Vanderbylia robiniophila* was collected from Liaoning Province, China, in August 2020 and authenticated based on the morphology analysis and ITS gene sequencing (GenBank Accession number OR116090) (Figure S33) [2]. The fungus was preserved at the Institute of Applied Ecology, Chinese Academy of Sciences, Shenyang.

### 2.4. Fermentation and Extraction

*Vanderbylia robiniophila* was cultured on malt extract agar (MEA) plates at 28 °C for 7 days. Then, 5 pieces of mycelia were inoculated in a 250 mL conical flask containing 150 mL liquid medium (3% malt extract) and shaking cultured at 28 °C for 7 days at 140 rpm. Afterward, the seed liquid culture (8 mL) was transferred into rice medium (100 g rice and 100 mL water) and fermented at 28 °C statically for 3 months. Subsequently, the cultures were extracted by 90% EtOH 5 times at room temperature. The residue was suspended in water and partitioned with EtOAc.

### 2.5. Purification

The EtOAc layer (214 g) was subjected to silica gel CC (200–300 mesh) and stepped gradient elution with petroleum ether-EtOAc (*v*/*v*, 50:1, 20:1, 10:1, 5:1, 1:1) and CH_2_Cl_2_-CH_3_OH (*v*/*v*, 100:1, 50:1, 30:1, 20:1, 10:1, 5:1, 1:1) to yield fractions A–D. Fr. B (9.5 g) was chromatographed using RP-C18 silica gel (EtOH-H_2_O, 10–90%) to obtain 5 fractions (Fr.B1–Fr.B5). Fr. B1 (2.5 g) was submitted to silica gel CC (petroleum ether-EtOAc, 50:1-1:1) and separated by HPLC (90.0% CH_3_CN-H_2_O) to afford **3** (5.5 mg, *t_R_* = 33.8 min). Fr. B2 (1.3 g) was purified by Sephadex LH-20 CC (CH_3_OH) and separated by HPLC (78.0% CH_3_CN-H_2_O) to afford **6** (3.0 mg, *t_R_* = 33.8 min) and **7** (2.1 mg, *t_R_* = 25.4 min). Fr. B5 (1.2 g) was divided into 4 subfractions (Fr. B5-1~Fr. B5-4) by silica gel with the mobile phase CH_2_Cl_2_-CH_3_OH (from 50:1 to 1:1). Fr. B5-1, B5-3, B5-4 were isolated by HPLC (CH_3_CN-H_2_O: 38.0%, 38.0%, 30.0%) to give compounds **8** (3.3 mg, *t_R_* = 57.6 min), **9** (1.0 mg, *t_R_* = 43.2 min), and **10** (2.8 mg, *t_R_* = 27.3 min). Fr.D (40 g) was separated on a glass column packed with HP-20 macroporous resin and eluted with increasing concentrations of EtOH in water to afford 3 subfractions (Fr.D1~Fr.D3). Fr. D1 (4.5 g), which was eluted by petroleum ether-EtOAc (*v*/*v*, 50:1, 30:1, 20:1, 10:1, 5:1, 1:1), was further purified using silica gel CC to obtain 3 subfractions (Fr. D1-1~Fr. D1-3). Compounds **1** (3.0 mg, *t_R_* = 70.0 min) and **2** (4.9 mg, *t_R_* = 44.2 min) were isolated from Fr. D1-1 (320.5 mg) via semipreparative HPLC (90.0% CH_3_CN-H_2_O). Compounds **4** (5.3 mg, *t_R_* = 34.1 min) and **5** (2.8 mg, *t_R_* = 51.7 min) were separated from Fr. D1-3 (200.7 mg) by semipreparative HPLC (78.0% CH_3_CN-H_2_O).

### 2.6. Spectroscopic Data

#### 2.6.1. Novel Compounds

*(22E, 24R)-6β, 7α-dimethoxyergosta-8(14), 22-diene-3β, 5α-diol* (**1**): White amorphous solid powder, [*α*${]}_{D}^{20}$ −227.78 (*c* 0.1, MeOH); UV (MeOH) λ_max_ (log *ε*): 238 (+1.09); IR (MeOH) _υmax_: 3680, 2935, 2869, 1455, 1369, 1033, 1013, 971 cm^−1^; ^1^H and ^13^C NMR data, Table 1; HRESIMS *m*/*z* 497.3616 [M + Na]^+^ (calcd for C_30_H_50_O_4_Na, 497.3607). (Figures S1–S9).

Vanderbyliolide A (**8**): Colorless transparent oil, [*α*${]}_{D}^{20}$ −744.44 (*c* 0.1, MeOH); UV (MeOH) λ_max_ (log *ε*): 235 (+1.28); IR (MeOH) _υmax_: 3680, 2923, 2858, 1737, 1436, 1381, 1261, 1033, 960, 899, 765 cm^−1^; ^1^H and ^13^C NMR data, Table 1; HRESIMS *m*/*z* 321.1697 [M + Na]^+^ (calcd for C_16_H_26_O_5_Na, 321.1678). (Figures S22–S28).

#### 2.6.2. Known Compounds

(22*E*, 24*R*)-6*β*-methoxyergosta-7, 9(11), 22-triene-3*β*, 5*α*-diol (**2**): ^1^H NMR (600 MHz, CDCl_3_) *δ*_H_ 5.71 (1H, d, *J* = 6.9, 2.1 Hz), 5.52 (1H, d), 5.24 (1H, dd, *J* = 15.3, 7.6 Hz), 5.17 (1H, dd, *J* = 15.3, 8.3 Hz), 4.10 (1H, m), 3.42 (3H, s), 3.34 (1H, dd, *J* = 5.6, 2.0 Hz), 2.35 (1H, m), 2.27 (1H, m), 2.15 (1H, m), 2.05 (1H, o), 2.05 (1H, o), 1.95 (1H, m), 1.86 (1H, m), 1.80 (1H, o), 1.80 (1H, o), 1.80 (1H, o), 1.80 (1H, o), 1.74 (1H, m), 1.56 (1H, m), 1.48 (1H, o), 1.48 (1H, o), 1.34 (1H, o), 1.34 (1H, o), 1.21 (3H, s), 1.02 (3H, d, *J* = 6.6 Hz), 0.92 (3H, d, *J* = 6.8 Hz), 0.84 (3H, d, *J* = 6.8 Hz), 0.83 (3H, d, *J* = 6.9 Hz), 0.60 (3H, s) ppm; ^13^C NMR (150 MHz, CDCl_3_) *δ*_C_ 140.6, 138.9, 135.5, 132.4, 126.0, 116.3, 82.9, 75.7, 67.9, 58.5, 56.2, 51.7, 43.0, 42.7, 42.5, 41.0, 40.5, 38.4, 33.2, 31.3, 31.1, 28.9, 26.0. 23.3, 20.8, 20.1, 19.8, 17.8, 11.6 ppm. (Figures S10 and S11).

5*α*, 6*α*-epoxy-3*β*-hydroxy-(22*E*)-ergosta-8(14), 22-dien-7-one (**3**): ^1^H NMR (600 MHz, CDCl_3_) *δ*_H_ 5.24 (1H, m), 5.19 (1H, dd, *J* = 15.3, 7.9 Hz), 3.92 (1H, tt, *J* = 11.3, 4.6 Hz), 3.16 (1H, s), 2.72 (2H, o), 2.72 (2H, o), 2.24 (1H, dd, *J* = 13.4, 11.6 Hz), 2.14 (1H, m), 2.01 (1H, o), 2.01 (1H, o), 1.87 (1H, m), 1.80 (1H, m), 1.75 (1H, m), 1.59 (1H, o), 1.59 (1H, o), 1.48 (1H, o), 1.48 (1H, o), 1.48 (1H, o), 1.48 (1H, o), 1.48 (2H, o), 1.24 (1H, m), 1.04 (3H, d, *J* = 6.7 Hz), 0.92 (3H, m), 0.92 (3H, m), 0.84 (3H, m), 0.82 (3H, m) ppm; ^13^C NMR (150 MHz, CDCl_3_) *δ*_C_ 195.6, 171.4, 134.9, 132.8, 124.0, 68.9, 68.9, 63.0, 54.7, 46.2, 43.0, 41.4, 39.2, 39.0, 36.4, 35.7, 33.2, 32.9, 31.2, 30.7, 27.9, 21.5, 20.1, 19.8, 19.6, 19.2, 17.8, 16.3 ppm. (Figures S12 and S13).

3*β*, 5*α*, 9*α*-trihydroxy- (22*E*, 24*R*)-ergosta-7, 22-dien-6-one (**4**): ^1^H NMR (600 MHz, CDCl_3_) *δ*_H_ 5.62 (1H, s), 5.23 (1H, dd, *J* = 15.2, 7.6 Hz), 5.16 (1H, dd, *J* = 15.2, 8.4 Hz), 4.06 (1H, m), 2.76 (1H, m), 2.34 (1H, m), 2.10 (1H, m), 1.94 (1H, m), 1.94 (1H, m), 1.87 (1H, o), 1.87 (1H, o), 1.80 (1H, m), 1.74 (1H, o), 1.74 (1H, o), 1.74 (1H, o), 1.58 (1H, m), 1.47 (1H, o), 1.47 (1H, o), 1.47 (1H, o), 1.47 (1H, o), 1.35 (1H, m), 1.03 (1H, d, *J* = 6.6 Hz), 0.99 (3H, s), 0.92 (3H, d, *J* = 6.8 Hz), 0.84 (3H, d, *J* = 6.8 Hz), 0.82 (3H, d, *J* = 6.8 Hz), 0.61 (3H, s) ppm; ^13^C NMR (150 MHz, CDCl_3_) *δ*_C_ 198.5, 165.1, 135.2, 132.6, 119.9, 79.7, 74.8, 67.4, 56.1, 51.9, 45.5, 43.0, 41.9, 40.4, 37.0, 35.1, 33.2, 30.1, 28.8, 28.0, 25.7, 22.6, 21.2, 20.5, 20.1, 19.8, 17.8, 12.4 ppm. (Figures S14 and S15).

(22*E*, 24*R*)-ergost-7, 22-dien-3*β*, 5*α*-diol-6-one (**5**): ^1^H NMR (600 MHz, CDCl_3_) *δ*_H_ 5.65 (1H, s), 5.24 (1H, dd, *J* = 15.3, 7.7 Hz), 5.16 (1H, dd, *J* = 15.3, 8.5 Hz), 4.04 (1H, m), 2.52 (1H, ddd, *J* = 12.1, 6.9, 2.5 Hz), 2.12 (1H, o), 2.12 (1H, o), 2.12 (1H, o), 2.04 (1H, o), 2.04 (1H, o), 1.87 (1H, m), 1.78 (1H, o), 1.78 (1H, o), 1.73 (1H, o), 1.73 (1H, o), 1.62 (1H, o), 1.62 (1H, o), 1.62 (1H, o), 1.62 (1H, o), 1.47 (1H, o), 1.47 (1H, o), 1.47 (1H, o), 1.36 (1H, m), 1.25 (1H, m), 1.03 (3H, d, *J* = 6.6 Hz), 0.95 (3H, s), 0.92 (3H, d, *J* = 6.8 Hz), 0.84 (3H, d, *J* = 6.8 Hz), 0.82 (3H, d, *J* = 6.8 Hz), 0.60 (3H, s) ppm; ^13^C NMR (150 MHz, CDCl_3_) *δ*_C_ 198.4, 165.4, 135.2, 132.7, 119.9, 78.0, 67.6, 56.2, 56.0, 44.9, 44.0, 43.0, 40.6, 40.4, 39.0, 36.7, 33.2, 30.5, 30.4, 28.0, 22.6, 22.1, 21.3, 20.1, 19.8, 17.7, 16.6, 12.8 ppm. (Figures S16 and S17).

Dankasterone (**6**): ^1^H NMR (600 MHz, CDCl_3_) *δ*_H_ 6.36 (1H, s), 5.27 (1H, m), 5.26 (1H, m), 2.81 (1H, t, *J* = 9.1 Hz), 2.66 (1H, d, *J* = 16.9, 1.5 Hz), 2.51 (1H, o), 2.51 (1H, o), 2.47 (2H, o), 2.47 (1H, o), 2.42 (1H, m), 2.03 (2H, o), 2.03 (1H, o), 1.87 (1H, o), 1.87 (1H, o), 1.87 (1H, o), 1.77 (1H, m), 1.71 (1H, o), 1.71 (1H, o), 1.48 (1H, o), 1.48 (1H, o), 1.26 (3H, s), 1.09 (3H, d, *J* = 7.0 Hz), 0.98 (3H, s), 0.91 (3H, d, *J* = 6.8 Hz), 0.83 (3H, d, *J* = 6.7 Hz), 0.81 (3H, d, *J* = 6.8 Hz) ppm; ^13^C NMR (150 MHz, CDCl_3_) *δ*_C_ 214.9, 200.2, 199.3, 156.2, 135.3, 132.5, 126.7, 62.3, 54.1, 49.5, 49.5, 43.4, 41.0, 39.1, 38.5, 38.1, 37.4, 36.2, 34.5, 33.2, 25.3, 24.2, 23.8, 23.3, 20.2, 19.8, 17.8, 17.2 ppm. (Figures S18 and S19).

4-hydroxy-17*R*-methylincisterol (**7**): ^1^H NMR (600 MHz, CDCl_3_) *δ*_H_ 5.64 (1H, d, *J* = 1.8 Hz), 5.26 (1H, d, *J* = 15.3, 8.5 Hz), 5.17 (1H, d, *J* = 15.3, 7.8 Hz), 2.64 (1H, m), 2.28 (1H, ddd, *J* = 14.2, 4.2, 2.4 Hz), 2.06 (1H, m), 1.98 (1H, ddd, *J* = 13.4, 4.8, 2.5 Hz), 1.90 (1H, m), 1.88 (1H, m), 1.85 (1H, m), 1.73 (1H, m), 1.63 (1H, m), 1.49 (1H, o), 1.49 (1H, o), 1.49 (1H, o), 1.49 (1H, o), 1.04 (3H, d, *J* = 6.7 Hz), 0.92 (3H, d, *J* = 6.8 Hz), 0.84 (3H, d, *J* = 6.8 Hz), 0.83 (3H, d, *J* = 6.8 Hz), 0.61 (3H, s) ppm; ^13^C NMR (150 MHz, CDCl_3_) *δ*_C_ 170.9, 170.6, 134.8, 133.0, 112.5, 104.9, 55.5, 50.5, 49.0, 43.0, 40.3, 35.4, 35.2, 33.2, 29.0, 21.5, 21.2, 20.1, 19.8, 17.7, 11.9 ppm. (Figures S20 and S21).

Cornilkone C (**9**): [*α*${]}_{D}^{20}$ -723.55 (*c* 0.1, MeOH); ^1^H NMR (600 MHz, CDCl_3_) *δ*_H_ 7.44 (1H, dd, *J* = 5.7, 1.5 Hz), 6.11 (1H, dd, *J* = 5.7, 2.0 Hz), 5.03 (1H, ddd, *J* = 7.2, 5.3, 2.7 Hz), 3.67 (3H, s), 2.30 (2H, t, *J* = 7.5 Hz), 1.76 (1H, m), 1.64 (2H, o), 1.64 (2H, o), 1.31 (2H, o), 1.31 (2H, o), 1.31 (2H, o), 1.31 (2H, o) ppm; ^13^C NMR (150 MHz, CDCl_3_) *δ*_C_ 174.4, 173.3, 156.4, 121.7, 83.5, 51.6, 34.2, 33.3, 29.2, 29.1, 29.1, 25.0, 25.0 ppm. (Figures S29 and S30).

(1*H*-indol-3-yl) oxoacetic acid methyl ester (**10**): ^1^H NMR (600 MHz, CDCl_3_) *δ*_H_ 8.51 (1H, d, *J* = 3.2 Hz), 8.46 (1H, m), 7.45 (1H, m), 7.36 (1H, m), 7.34 (1H, m), 3.96 (3H, s) ppm; ^13^C NMR (150 MHz, CDCl_3_) *δ*_C_ 177.8, 163.3, 136.6, 136.1, 126.2, 124.7, 123.8, 122.8, 114.6, 111.7, 52.9 ppm. (Figures S31 and S32).

### 2.7. ECD Calculations

The conformational searches were carried out employing SPARTAN 14 in the MMFF force field. The conformers under the 3.0 kJ/mol energy window were further optimized at the B3LYP/6-31G(d) level by DFT using the Gaussian 09 package [29]. The optimized stable conformers were calculated using TDDFT methods at the B3LYP/6-311+g(d,p) level with an implicit solvent model for CDCl_3_. Then, the calculated ECD curve weighted by the Boltzmann distribution was produced by SpecDis 1.71 [30].

### 2.8. Bioassays

#### 2.8.1. Anti-Tyrosinase Activities

The inhibitory effects of the isolated compounds on the tyrosinase were determined according to the literature procedure with some modifications [31]. L-tyrosine solution was used as substrate in concentration of 2 mM (40 uL). Compounds **1–10** (40 μL) with increased concentrations (5, 10, 50, 100, 200 μM), tyrosinase (40 μL, 100 U/mL), and 80 µL of phosphate-buffered saline (PBS) solution (25 mM, pH 6.8) were added to a 96-well microplate. A quantity of 40 μL 20% MeOH solution took the place of the sample as a blank solution. The mixtures were incubated at 37 °C for 30 min. After incubation, the produced dopachrome was measured at 492 nm by a microplate reader. The inhibition ratio was calculated by [1 − (C − D)/(A − B) × 100%], where A is the absorbance of blank solution after incubation, B is the absorbance of blank solution before incubation, C is the absorbance of sample solution after incubation, and D is the absorbance of sample solution before incubation. The IC_50_ values and statistical analyses were performed using GraphPad Prism 7 software, and the results were expressed as means ± SD of triplicate determination.

#### 2.8.2. Kinetic Analysis

The inhibition type was measured by the reaction rate–substrate concentration curve and the Lineweaver–Burk plot. L-tyrosine solutions were diluted to different concentrations (5 mM, 7.5 mM, 9 mM, 12 mM, 15 mM) as the substate. The inhibition constant was determined by the second plot of the y-intercept versus the concentration of the inhibitor. The values of *K_is_* were calculated from the following formula:

$$y\text{-}\mathrm{intercept}=\frac{1}{V_{m}}\left(1+\frac{\left[I\right]}{K_{is}}\right)$$

## 3. Results and Discussion

### 3.1. Structure Elucidation

**Figure 2:**
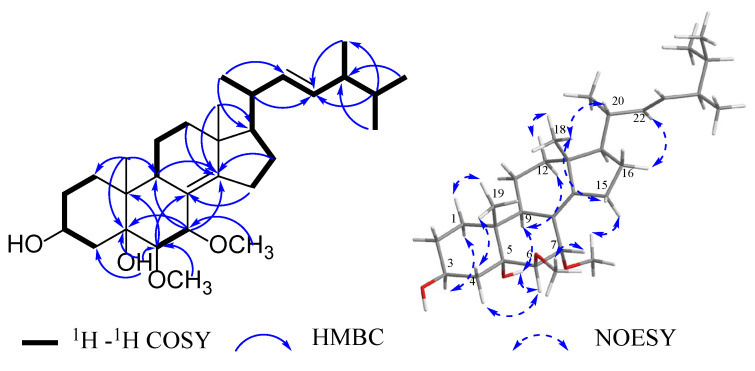
Key ^1^H-^1^H COSY, HMBC and NOESY correlations of compound **1**.

Compound **1**, a white amorphous solid powder, was assigned the molecular formula C_30_H_50_O_4_ by analyses of HRESIMS *m*/*z* 497.3616 [M + Na]^+^ (calcd for C_30_H_50_O_4_Na, 497.3607) and NMR data (Table 1). ^1^H NMR spectrum of **1** revealed characteristic signals for two methyl singlets at *δ*_H_ 0.91 (H-18 and H-19 with the specific data *δ*_H_ 0.911 and 0.907), four methyl doublets at *δ*_H_ 1.05 (3H, *J* = 6.7 Hz, H-21), 0.84 (3H, *J* = 6.8 Hz, H-26), 0.83 (3H, *J* = 6.7 Hz, H-27) and 0.93 (3H, *J* = 6.8 Hz, H-28), two disubstituted double bonds at *δ*_H_ 5.19 (1H, dd, *J* = 15.3, 8.2 Hz, H-22), 5.25 (1H, dd, *J* = 15.3, 7.3 Hz, H-23), a sp^3^ oxygenated methine group at *δ*_H_ 4.11 (1H, tt, *J* = 11.0, 5.2 Hz, H-3), and two methoxy groups at *δ*_H_ 3.36 (3H, s, 6-OCH_3_), 3.21 (3H, s, 7-OCH_3_). The ^13^C NMR data (Table 1) showed resonances for four olefinic carbons [*δ*_C_ 122.2 (C-8), 132.5 (C-14), 135.4 (C-22), 153.6 (C-23)], four oxygenated carbons [*δ*_C_ 67.5 (C-3), 76.4 (C-7), 76.8 (C-5), 85.7 (C-6)], two methoxy groups (*δ*_C_ 54.7, 59.5) and the other 22 signals for aliphatic carbons. The above results indicated that the planar structure of **1** was most likely an ergostane-type steroid. The ^1^H-^1^H COSY correlations of H-1/H-2/H-3/H-4, H-6/H-7, H-9/H-11/H-12, together with the HMBC correlations of H-6/C-8, 10, H-7/C-5, 9, 14, H-9, 12, 16, 18/C-14, H-19/C-1, 5, 9, and methoxyl protons/C-6, 7 (Figure 2) further verified the steroidal tetracyclic skeleton of **1** with two hydroxy groups located at C-3 and C-5, two methoxyl groups linked to C-6 and C-7, and a Δ^8(14)^ double bond. A long spin-coupling system from H-15 to H-28 recognized in the ^1^H-^1^H COSY spectrum, combined with the HMBC correlations of H-26,27/C-24, H-25/C-23,28, H-20,28/C-23, H-21/C-17,22, established the alkyl side chain at C-17 with a trans double bond between C-22 and C-23.

The relative configuration of **1** was elucidated as shown on the basis of a NOESY experiment (Figure 2). The *α*-orientation of H-3 and H-6 was ascertained by the cross-peaks in sequences of H-19/H-1*α*, 4*α*, H-3/H-1*β*, and H-4*α*/H-6/5-OH/H-10, while the *β*-orientation of H-7 was evidenced by the correlation of H-7/6-OCH_3_. The observation of NOE signals between H-18 and H-12*β*, 15*β*, 20, H-22 and H-16, 7-OCH_3_, and H-15*α* was consistent with the *α*-oriented H-17 and the 20R* stereoisomer. The 24*R* configuration was determined by comparing the ^13^C NMR resonance peaks of C-28 (*δ*_C_ 17.4) in CDCl_3_, which should be about 0.4 ppm downfield shifted for C-28 in the 24*S* epimer [32,33]. The above data showed that **1** was a 6,7-OCH_3_ substituted ergostane-type steroid compared to the known compound [34]. Therefore, the structure of **1** was identified as (22*E*, 24*R*)-6*β*, 7*α*-dimethoxyergosta-8(14), 22-diene-3*β*, 5*α*-diol.

**Figure 3:**
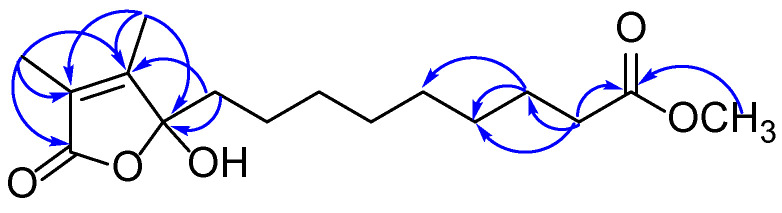
Key HMBC correlations of compound **8**.

**Figure 4:**
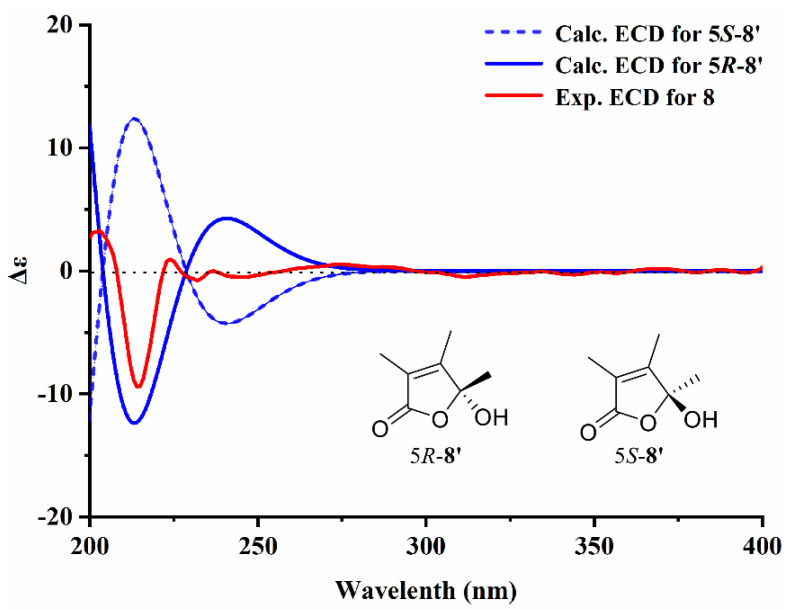
Calculated and experimental ECD spectra for **8**.

Compound **8** was isolated as a transparent oil with the chemical formula of C_16_H_26_O_5_ deduced from HRESIMS *m*/*z* 321.1697 [M + Na]^+^ (calcd for C_16_H_26_O_5_Na, 321.1678). Analysis of the 1D and 2D NMR spectra of **8** revealed that a sets of long-range coupling methyls [*δ*_H_ 1.82 (3H, d, *J* = 1.2 Hz, H-15), 1.94 (3H, d, *J* = 1.2 Hz, H-16) and *δ*_C_ 8.6 (C-15), 10.8 (C-16)], two olefinic carbons [*δ*_C_ 157.7 (C-3) and 125.5 (C-4)], an ester carbonyl [*δ*_C_ 172.0 (C-2)] and an oxygenated quaternary carbon [*δ*_C_ 106.9 (C-5)] formed a *α*, *β*-unsaturated 2,3-dimethyl-*γ*-lactone moiety by key HMBC correlations from H-15 to C-2, 4 and from H-16 to C-3, 5 (Figure 3). The monocyclic system was further confirmed by the four degrees of unsaturation. Moreover, a methyl nonanoate side chain was established by the signals of methylene groups at *δ*_H_ 2.29 (2H, t, *J* = 7.5 Hz, H-13), 1.97 (1H, m, H-6a), 1.75 (1H, m, H-6b), 1.60 (1H, m, H-12), 1.17–1.27 (10H, overlap, H-1~11) and terminal COOCH_3_ unit at *δ*_H_ 3.66 (3H, s, 14-OCH_3_) and *δ*_C_ 174.5 (C-14), as well as the HMBC correlations from 14-OCH_3_ and H-12, 13 to C-14. It was directly connected to C-5 in the lactonic ring deciphered by the key interactions from H-6 to C-5 in the HMBC spectrum. Given the flexible aliphatic chain could produce excess conformations, the calculated ECD method was applied on a simplified model compound to determine the absolute configuration of **8** at B3LYP/6-31G (d, p) level in MeOH (Figure 4). As a result, the experimental Cotton effects of **8** were in agreement with the calculated Cotton effects for 5*S*-**8**′. These data were similar to the known compound caulerpalide A [35]; the only difference between them is that an ethoxy group at position C-14 in caulerpalide A was substituted by a methoxy group in **8**. Thus, compound **8** was established as shown and named vanderbyliolide A.

Eight known compounds were identified as (22*E*, 24*R*)-6*β*-methoxyergosta-7, 9(11), 22-triene-3*β*, 5*α*-diol (**2**) [36], 5*α*,6*α*-epoxy-3*β*-hydroxy-(22*E*)-ergosta-8(14),22-dien-7-one (**3**) [37], 3*β*, 5*α*, 9*α*-trihydroxy-(22*E*, 24*R*)-ergosta-7, 22-dien-6-one (**4**) [38], (22*E*, 24*R*)-ergost-7, 22-dien-3*β*, 5*α*-diol-6-one (**5**) [39], dankasterone (**6**) [40], 4-hydroxy-17*R*-methylincisterol (**7**) [41], cornilkone C (**9**) [42], and (1*H*-indol-3-yl) oxoacetic acid methyl ester (**10**) [43] by comparing their spectroscopic data with the literature. All compounds were isolated from the species of the genus *Vanderbylia* for the first time. Among them, cornilkone C (**9**) was first isolated from fungi. It was originally discovered and purified from corn silk as a pair of enantiomeric compounds with weak anti-A*β*_1–42_ aggregation activity [42]. Notably, the type of 2(5*H*)-furanone derivatives (**8–9**) in our study exhibited significant specific rotations, which are significantly different from the minor magnitudes in the literature. It was indicated that **8** and **9** derived from fungi were likely to be optical pure compounds or racemic mixtures in different proportions. Compound **10** had been obtained from marine-derived fungi with weak cytotoxic activity against HeLa cells [44,45,46]. It was detected from macrofungi for the first time in this study.

### 3.2. Anti-Tyrosinase Activities

Tyrosinase inhibitors have been clinically used for diseases associated with melanin hyperpigmentation [47]. In this study, the inhibitory effects of **1–10** on tyrosinase were determined spectrophotometrically compared to the positive control arbutin (Table 2). Among them, compound **10** showed the highest tyrosinase inhibitory activity with IC_50_ values of 60.47 ± 2.63 μM, which were comparable to those of arbutin (IC_50_ = 58.17 ± 6.09 μM). Compounds **2**, **4**, **5**, and **8** exhibited weak inhibitory activities with IC_50_ values ranging from 94.16 to 148.38 μM. The remaining compounds did not show any tyrosinase inhibitory activity at the texted concentration. These observations demonstrated that the indole alkaloid exhibited much stronger inhibitory potency than the other structures. It has been reported that the amino groups such as an indole ring could improve the tyrosinase inhibitory activity, which was consistent with our results [48,49]. Among the isolated steroids, compound **6** has a structural feature of C-ring migration and compound **7** is a highly degraded sterol belonging to the class incisterols. They were inactive even at 200 μM, indicating the importance of the tetracyclic ergostane-type scaffold. Detailed inspection of the structures of compounds **2**, **4**, and **5** showed the common features of a double bond at Δ^7(8)^ within these molecules, which indicated that the position of olefinic bond is crucial for the activity.

### 3.3. Enzyme Kinetic Analysis

**Figure 5:**
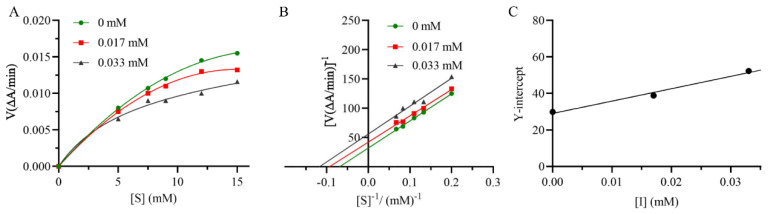
Inhibition mechanism on tyrosinase of compound **10**: (**A**) reaction rate–substrate concentration curve; (**B**) Lineweaver–Burk plots; (**C**) the secondary replots of y-intercept vs. [inhibitor].

To confirm the inhibition mechanism of the most potent compound (**10**) on the inhibitory activity of tyrosinase, the V-S and Lineweaver–Burk plots were constructed. As shown in Figure 5A,B, both *K*_m_ and *V*_m_ values of **10** decreased with the increase in concentration, but the ratio of *K*_m_/*V*_m_ remained unchanged. Thus, **10** belongs to an uncompetitive inhibitor, demonstrating that it inhibits the enzyme by combining with the enzyme–substrate complex. The inhibition constant *K*_is_ was obtained from the plot of the y-intercept versus the concentration of **10**, which was calculated to be 0.04 mM (Figure 5C).

## 4. Conclusions

In summary, two new compounds, (22*E*, 24*R*)-6*β*, 7*α*-dimethoxyergosta-8(14), 22-diene-3*β*, 5*α*-diol (**1**) and vanderbyliolide A (**8**), together with eight known ones (**2–7**, **9–10**), were isolated from the cultures of *Vanderbylia robiniophila*. All compounds were discovered from the genus for the first time. Compound **10** showed potential tyrosinase inhibitory activity comparable to that of arbutin. The kinetics of the enzymatic reaction indicated that **10** was an uncompetitive inhibitor on tyrosinase. This study provides evidence for the development and utilization of *V. robiniophila* in skin disorders associated with melanin hyperpigmentation.

## Figures and Tables

**Table 1:** The ^1^H (600 MHz) and ^13^C NMR (150 MHz) Data ^a^ of **1** and **8** in CDCl_3_.

1 (*δ* in ppm, Multi, *J* in Hz)						8 (*δ* in ppm, Multi, *J* in Hz)		
No.	*δ*_H_	*δ*_C_	No.	*δ*_H_	*δ*_C_	No.	*δ*_H_	*δ*_C_
1	1.75 (m) 1.29 (m)	31.7	16	1.43 (o) 1.73 (m)	27.6	2	-	172.0
2	1.43 (o) 1.86 (m)	31.1	17	1.19 (m)	57.3	3	-	125.5
3	4.11 (tt, 11.0, 5.2)	67.5	18	0.91 (s)	17.7	4	-	157.7
4	1.69 (m) 1.95 (m)	40.6	19	0.91 (s)	17.8	5	-	106.9
5	-	76.8	20	2.14 (m)	39.4	6	1.75 (m) 1.97 (m)	36.2
6	3.12 (d, 2.3)	85.7	21	1.05 (d, 6.7)	21.5	7	1.17 (m) 1.27 (o)	23.0
7	4.22 (d, 2.3)	76.4	22	5.19 (dd, 15.3, 8.2)	135.4	8	1.27 (o)	29.4
8	-	122.2	23	5.25 (dd, 15.3, 7.3)	132.5	9	1.27 (o)	29.3
9	2.33 (m)	36.7	24	1.86 (m)	43.0	10	1.27 (o)	29.2
10	-	41.1	25	1.48 (m)	33.2	11	1.27 (o)	29.2
11	1.53 (m) 1.57 (m)	19.3	26	0.84 (d, 6.8)	20.1	12	1.60 (m)	25.0
12	1.16 (m) 2.00 (m)	37.1	27	0.83 (d, 6.7)	19.8	13	2.29 (t, 7.5)	34.2
13	-	43.9	28	0.93 (d, 6.8)	17.4	14	-	174.5
14	-	153.6	5-OH	4.40 (s)	-	15	1.82 (d, 1.2)	8.6
15	2.32 (m) 2.40 (m)	25.8	6-OCH_3_	3.36 (s)	59.5	16	1.94 (d, 1.2)	10.8
			7-OCH_3_	3.21 (s)	54.7	14-OCH_3_	3.66 (s)	51.6

^a ^Overlapping signals are expressed as o.

**Table 2:** The inhibitory effects of **1–10** on tyrosinase.

Compounds	IC_50_ (μM)	Compounds	IC_50_ (μM)
**1**	>200	**6**	>200
**2**	148.38 ± 23.67	**7**	>200
**3**	>200	**8**	102.53 ± 4.05
**4**	116.36 ± 13.45	**9**	>200
**5**	94.16 ± 13.69	**10**	60.47 ± 2.63
Arbutin	58.17 ± 6.09		

## Data Availability

All data generated or analyzed in this study are included in manuscript and Supplementary Materials files.

## Supplementary Materials

The following supporting information can be downloaded at: https://www.mdpi.com/article/10.3390/jof9070702/s1, Figures S1–S32: The spectrum of compounds **1–10**; Figure S33: Basidiomes of *Vanderbylia robiniophila*.

## References

[1] Pavic A., Ilic-Tomic T., Glamočlija J. (2021). Unravelling anti-melanogenic potency of edible mushrooms *Laetiporus sulphureus* and *Agaricus silvaticus* in vivo using the zebrafish model. J. Fungi.

[2] Li Y.M., Huang J.H., Lu J.Y., Ding Y.F., Jiang L., Hu S.H., Chen J., Zeng Q.H. (2019). The role and mechanism of Asian medicinal plants in treating skin pigmentary disorders. J. Ethnopharmacol..

[3] Lee S.Y., Baek N., Nam T. (2016). Natural, semisynthetic and synthetic tyrosinase inhibitors. J. Enzym. Inhib. Med. Chem..

[4] Kishore N., Twilley D., Blom van Staden A., Verma P., Singh B., Cardinali G., Kovacs D., Picardo M., Kumar V., Lall N. (2018). Isolation of flavonoids and flavonoid glycosides from *Myrsine africana* and their inhibitory activities against mushroom tyrosinase. J. Nat. Prod..

[5] Pillaiyar T., Manickam M., Namasivayam V. (2017). Skin whitening agents: Medicinal chemistry perspective of tyrosinase inhibitors. J. Enzym. Inhib. Med. Chem..

[6] Caritá A.C., Fonseca-Santos B., Shultz J.D., Michniak-Kohn B., Chorilli M., Leonardi G.R. (2020). Vitamin C: One compound, several uses. Advances for delivery, efficiency and stability. Nanomedicine.

[7] Shim J.H. (2021). Inhibitory effects of cycloheterophyllin on melanin synthesis. Molecules.

[8] McDonald T.A., Holland N.T., Skibola C., Duramad P., Smith M.T. (2001). Hypothesis: Phenol and hydroquinone derived mainly from diet and gastrointestinal flora activity are causal factors in leukemia. Leukemia.

[9] Fogarasi M., Socaci S.A., Dulf F.V., Diaconeasa Z.M., Fărcaș A.C., Tofană M., Semeniuc C.A. (2018). Bioactive compounds and volatile profiles of five Transylvanian wild edible mushrooms. Molecules.

[10] Fogarasi M., Diaconeasa Z.M., Pop C.R., Fogarasi S., Semeniuc C.A., Fărcaş A.C., Țibulcă D., Sălăgean C.-D., Tofană M., Socaci S.A. (2020). Elemental composition, antioxidant and antibacterial properties of some wild edible mushrooms from Romania. Agronomy.

[11] Schueffler A., Anke T. (2014). Fungal natural products in research and development. Nat. Prod. Rep..

[12] Gressler M., Lohr N.A., Schafer T., Lawrinowitz S., Seibold P.S., Hoffmeister D. (2021). Mind the mushroom: Natural product biosynthetic genes and enzymes of Basidiomycota. Nat. Prod. Rep..

[13] Rittenhouse S., Biswas S., Broskey J., McCloskey L., Moore T., Vasey S., West J., Zalacain M., Zonis R., Payne D. (2006). Selection of retapamulin, a novel pleuromutilin for topical use. Antimicrob. Agents Chemother..

[14] Chiba K. (2020). Discovery of fingolimod based on the chemical modification of a natural product from the fungus, *Isaria sinclairii*. J. Antibiot..

[15] Pan J., Yang C.H., Jiang Z., Huang J. (2019). *Trametes robiniophila* Murr: A traditional Chinese medicine with potent anti-tumor effects. Cancer Manag. Res..

[16] Chen Q., Shu C., Laurence A.D., Chen Y., Peng B.G., Zhen Z.J., Cai J.Q., Ding Y.T., Li L.Q., Zhang Y.B. (2018). Effect of Huaier granule on recurrence after curative resection of HCC: A multicentre, randomised clinical trial. Gut.

[17] Li C., Wang X., Chen T., Wang W., Yang Q. (2020). *Trametes robiniophila* Murr in the treatment of breast cancer. Biomed. Pharm..

[18] Wang Y.X., Yuan H.S. (2021). Research progress on chemical constituents and anti-tumor effects of Huai’er (*Vanderbylia robiniophila*). Mycosystema.

[19] Lv F., Li X., Wang Y. (2022). An extraction from *Trametes robiniophila* Murr. (Huaier) inhibits non-small cell lung cancer proliferation via targeting to epidermal growth factor receptor. Bioengineered.

[20] Niu Y.J., Shan L., Gao H., Zhang C.C., Qian Z.J., Wang Z.X., Xu X., Zhang X., Wang J.Y., Ma L.F. (2020). Huaier suppresses the hepatocellular carcinoma cell cycle by regulating minichromosome maintenance proteins. OncoTargets Ther..

[21] Hu Z.D., Yang A.L., Su G.Z., Zhao Y.F., Wang Y., Chai X.Y., Tu P.F. (2016). Huaier restrains proliferative and invasive potential of human hepatoma SKHEP-1 cells partially through decreased Lamin B1 and elevated NOV. Sci. Rep..

[22] Li Y., Qi W., Song X., Lv S., Zhang H., Yang Q. (2016). Huaier extract suppresses breast cancer via regulating tumor-associated macrophages. Sci. Rep..

[23] Shan L., Li Y., Jiang H.Y., Tao Y.Q., Qian Z.J., Li L., Cai F., Ma L.F., Yu Y.C. (2017). Huaier restrains proliferative and migratory potential of hepatocellular carcinoma cells partially through decreased yes-associated protein 1. J. Cancer.

[24] Wang L.J., Yu Z.X., Wei C., Zhang L., Song H., Chen B., Yang Q.F. (2017). Huaier aqueous extract protects against dextran sulfate sodium-induced experimental colitis in mice by inhibiting NLRP3 inflammasome activation. Oncotarget.

[25] Luo Z.Y., Hu X.P., Xiong H., Qiu H., Yuan X.L., Zhu F., Wang Y.H., Zou Y.M. (2016). A polysaccharide from Huaier induced apoptosis in MCF-7 breast cancer cells via downregulation of MTDH protein. Carbohydr. Polym..

[26] Fang L., Zhang Y.Z., Zang Y.W., Chai R., Zhong G.X., Li Z.Y., Duan Z.C., Ren J.C., Xu Z.H. (2019). HP-1 inhibits the progression of ccRCC and enhances sunitinib therapeutic effects by suppressing EMT. Carbohydr. Polym..

[27] Jian T.T., Zhang Y., Zhang G.Y., Ling J.Y. (2022). Metabolomic comparison between natural Huaier and artificial cultured Huaier. Biomed. Chromatogr..

[28] Li K.X., Lv J.H., Dong J., Fan D.Y., Wang Y.P., Li C.T. (2022). Chemical constituents from the ethyl acetate of fermentation liquid in *Trames robiniophila*. Chin. Tradit. Pat. Med..

[29] Frisch M.J., Trucks G.W., Schlegel H.B., Scuseria G.E., Robb M.A., Cheeseman J.R., Scalmani G., Barone V., Mennucci B., Petersson G.A. (2013). The Gaussian 09 Package.

[30] Bruhn T., Schaumloffel A., Hemberger Y., Bringmann G. (2013). SpecDis: Quantifying the comparison of calculated and experimental electronic circular dichroism spectra. Chirality.

[31] Dai Y., Zhou G.X., Kurihara H., Ye W.C., Yao X.S. (2006). Biphenyl glycosides from the fruit of *Pyracantha fortuneana*. J. Nat. Prod..

[32] Yan X.H., Liu H.L., Huang H., Li B.X., Guo Y.W. (2011). Steroids with aromatic A-rings from the Hainan soft coral *Dendronephthya studeri* Ridley. J. Nat. Prod..

[33] Kikuchi T., Anami D., Morikawa S., Nakagawa Y., Yamada T., Li W., Hirano T. (2023). Secoergostane-and ergostane-type steroids from *Pleurotus cornucopiae* var. *citrinopileatus*. Phytochemistry.

[34] Sun Y., Tian L., Huang J., Li W., Pei Y.H. (2006). Cytotoxic sterols from marine-derived fungus *Pennicillium* sp.. Nat. Prod. Res..

[35] Li D.C., Yang Y., Zhang B., Liao X.J., Jiang Z.H., Xu S.H., Zhao B.X. (2020). Three New Butenolides from the Green Alga *Caulerpa racemosa* var. *turbinate*. Chem. Biodivers..

[36] Bao F.Y., Yang K.Y., Wu C.R., Gao S.Y., Wang P.H., Chen L.X., Li H. (2018). New natural inhibitors of hexokinase 2 (HK2): Steroids from *Ganoderma sinense*. Fitoterapia.

[37] Yaoita Y., Satoh Y., Kikuchi K. (2007). A new ceramide from *Ramaria botrytis* (Pers.) Ricken. J. Nat. Med..

[38] Wang H., Liu T.X., Xin Z.H. (2014). A new glucitol from an endophytic fungus *Fusarium equiseti* Salicorn. Eur. Food Res. Technol..

[39] Shang X.Y., Li J.J., Liu M.T., Li S., Liu Y., Wang Y.F., Huang X., Jin Z.L. (2011). Cytotoxic steroids from *Monascus purpureus*-fermented rice. Steroids.

[40] Amagata T., Tanaka M., Yamada T., Doi M., Minoura K., Ohishi H., Yamori T., Numata A. (2007). Variation in cytostatic constituents of a sponge-derived *Gymnascella dankaliensis* by manipulating the carbon source. J. Nat. Prod..

[41] Togashi H., Mizushina Y., Takemura M., Sugawara F., Koshino H., Esumi Y., Uzawa J., Kumagai H., Matsukage A., Yoshida S. (1998). 4-hydroxy-17-methylincisterol, an inhibitor of DNA polymerase-α activity and the growth of human cancer cells in vitro. Biochem. Pharm..

[42] Zhou W.Y., Lou L.L., Guo R., Xi Y.F., Lin B., Huang X.X., Song S.J. (2019). Diverse metabolites from corn silk with anti-Aβ_1-42_ aggregation activity. Fitoterapia.

[43] Bao B.Q., Zhang P., Lee Y., Hong J.K., Lee C., Jung J. (2007). Monoindole alkaloids from a marine sponge *Spongosorites* sp.. Mar. Drugs.

[44] Aiello A., Fattorusso E., Imperatore C., Irace C., Luciano P., Menna M., Santamaria R., Vitalone R. (2011). Zorrimidazolone, a bioactive alkaloid from the non-indigenous Mediterranean stolidobranch *Polyandrocarpa zorritensis*. Mar. Drugs.

[45] Longeon A., Copp B.R., Quevrain E., Roue M., Kientz B., Cresteil T., Petek S., Debitus C., Bourguet-Kondracki M. (2011). Bioactive indole derivatives from the South Pacific marine sponges *Rhopaloeides odorabile* and *Hyrtios* sp.. Mar. Drugs.

[46] Jennings L.K., Khan N.M.D., Kaur N., Rodrigues D., Morrow C., Boyd A., Thomas O.P. (2019). Brominated bisindole alkaloids from the celtic sea sponge *Spongosorites calcicole*. Molecules.

[47] Seiberg M., Paine C., Sharlow E., Andrade-Gordon P., Costanzo M., Eisinger M., Shapiro S.S. (2000). Inhibition of melanosome transfer results in skin lightening1. J. Investig. Dermatol..

[48] Zhai Y.J., Huo G.M., Zhang Q., Li D., Wang D.C., Qi J.Z., Han W.B., Gao J.M. (2020). Phaeosphaones: Tyrosinase inhibitory thiodiketopiperazines from an endophytic *Phaeosphaeria fuckelii*. J. Nat. Prod..

[49] Pillaiyar T., Namasivayam V., Manickam M., Jung S.H. (2018). Inhibitors of melanogenesis: An updated review. J. Med. Chem..

