# Supplementary material for: Secondary Metabolites from the Cultures of Medicinal Mushroom Vanderbylia robiniophila and Their Tyrosinase Inhibitory Activities
Source: J Fungi (Basel). 2023 Jun 26;9(7):702. doi: 10.3390/jof9070702 (PMC10381909; doi:10.3390/jof9070702)
Supplement: Supplementary file 1 [file jof-09-00702-s001.zip › jof-2373268-supplementary.pdf]

Novel secondary metabolites from the cultures of medicinal mushroom *Vanderbylia robiniophila* and their tyrosinase inhibitory activities

Yu-Xi Wang[1], Jing-Hui Jia[1, 2], Qi Wang[1, 3], Yu-Lian Wei[1], Hai-Sheng Yuan[1, 2, 3, *]

[1] CAS Key Laboratory of Forest Ecology and Management, Institute of Applied Ecology, Chinese Academy of Sciences, Shenyang 110164, China

[2] College of Life Sciences, Liaoning University, Shenyang 110036, China

[3] School of Life Sciences and Biopharmaceutics, Shenyang Pharmaceutical University, Shenyang 110016, China

* Correspondence: hsyuan@iae.ac.cn

Table of Contents

Figure S1. UV spectrum of compound **1**

Figure S2. IR spectrum of compound **1**

Figure S3. ^{1}H NMR spectrum (600 MHz, $CDCl_3$) of compound **1**

Figure S4. ^{13}C NMR spectrum (150 MHz, $CDCl_3$) of compound **1**

Figure S5. HMBC spectrum (600 MHz, $CDCl_3$) of compound **1**

Figure S6. HSQC spectrum (600 MHz, $CDCl_3$) of compound **1**

Figure S7. ^{1}H-^{1}H COSY spectrum (600 MHz, $CDCl_3$) of compound **1**

Figure S8. NOESY spectrum (600 MHz, $CDCl_3$) of compound **1**

Figure S9. HRESIMS spectrum of compound **1**

Figure S10. ^{1}H NMR spectrum (600 MHz, $CDCl_3$) of compound **2**

Figure S11. ^{13}C NMR spectrum (150 MHz, $CDCl_3$) of compound **2**

Figure S12. ^{1}H NMR spectrum (600 MHz, $CDCl_3$) of compound **3**

Figure S13. ^{13}C NMR spectrum (150 MHz, $CDCl_3$) of compound **3**

Figure S14. ^{1}H NMR spectrum (600 MHz, $CDCl_3$) of compound **4**

Figure S15. ^{13}C NMR spectrum (150 MHz, $CDCl_3$) of compound **4**

Figure S16. ^{1}H NMR spectrum (600 MHz, $CDCl_3$) of compound **5**

Figure S17. ^{13}C NMR spectrum (150 MHz, $CDCl_3$) of compound **5**

Figure S18. ^{1}H NMR spectrum (600 MHz, $CDCl_3$) of compound **6**

Figure S19. ^{13}C NMR spectrum (150 MHz, $CDCl_3$) of compound **6**

Figure S20. ^{1}H NMR spectrum (600 MHz, $CDCl_3$) of compound **7**

Figure S21. ^{13}C NMR spectrum (150 MHz, $CDCl_3$) of compound **7**

Figure S22. UV spectrum of compound **8**

Figure S23. IR spectrum of compound **8**

Figure S24. ^{1}H NMR spectrum (600 MHz, $CDCl_3$) of compound **8**

Figure S25. ^{13}C NMR spectrum (150 MHz, $CDCl_3$) of compound **8**

Figure S26. HMBC spectrum (600 MHz, $CDCl_3$) of compound **8**

Figure S27. HSQC spectrum (600 MHz, $CDCl_3$) of compound **8**

Figure S28. HRESIMS spectrum of compound **8**

Figure S29. ^{1}H NMR spectrum (600 MHz, $CDCl_3$) of compound **9**

Figure S30. ^{13}C NMR spectrum (150 MHz, $CDCl_3$) of compound **9**

Figure S31. ^{1}H NMR spectrum (600 MHz, $CDCl_3$) of compound **10**

Figure S32. ^{13}C NMR spectrum (150 MHz, $CDCl_3$) of compound **10**

Figure S33. Basidiomes of *Vanderbylia robiniophila*. Photo taken by Hai-Sheng Yuan in China, 2020

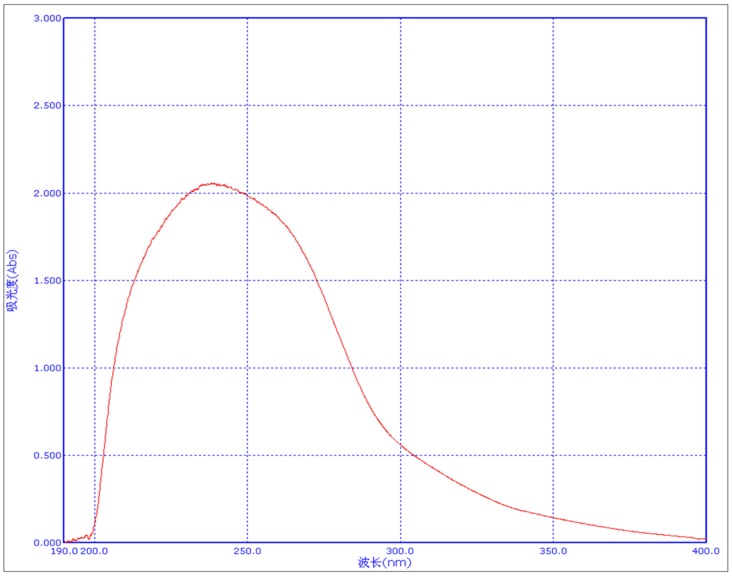

Figure S1. UV spectrum of compound **1**

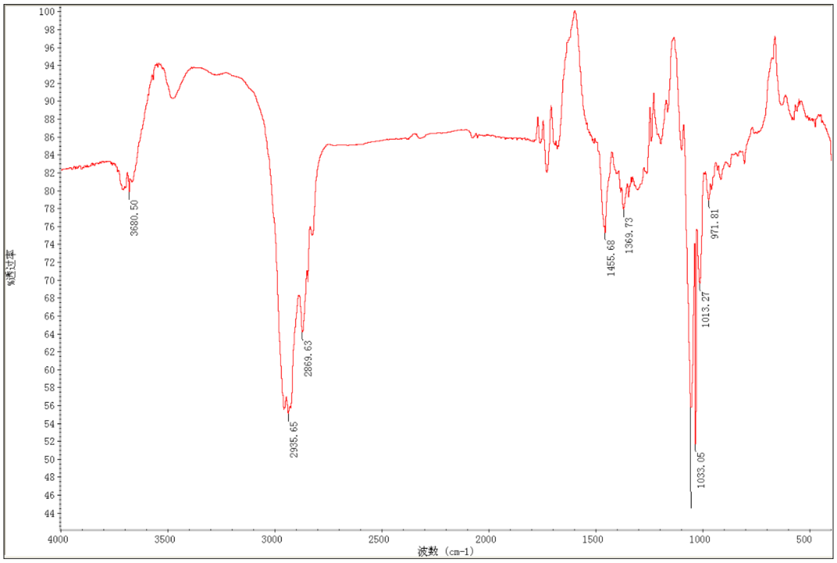

Figure S2. IR spectrum of compound **1**

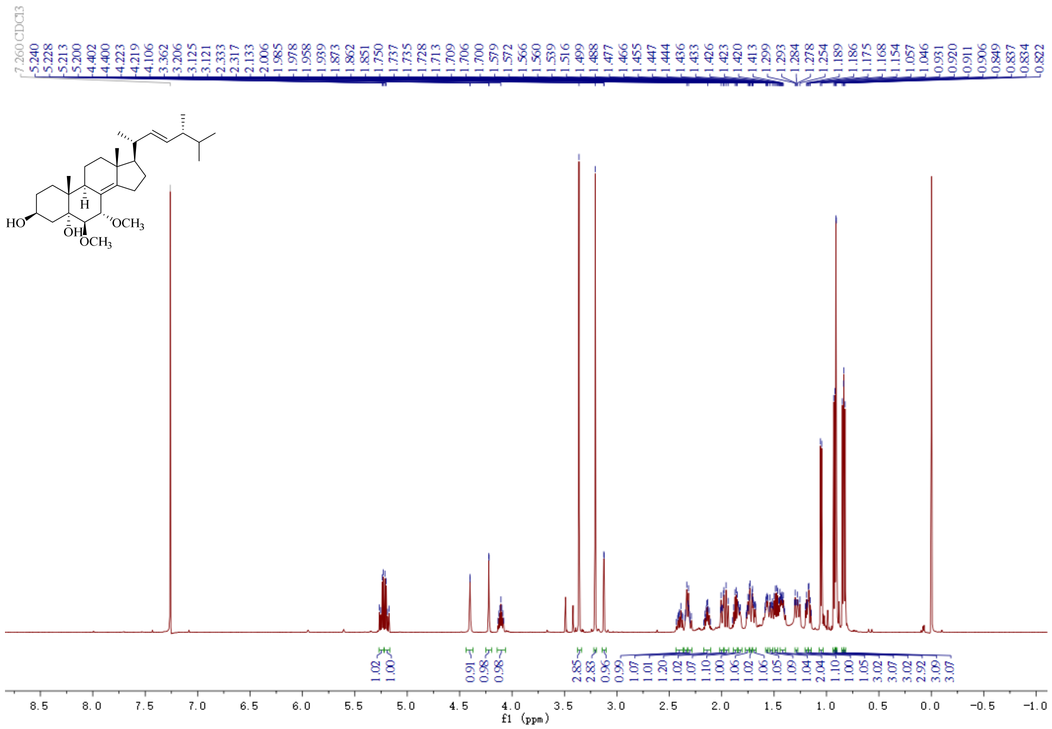

Figure S3. ^{1}H NMR spectrum (600 MHz, $CDCl_3$) of compound **1**

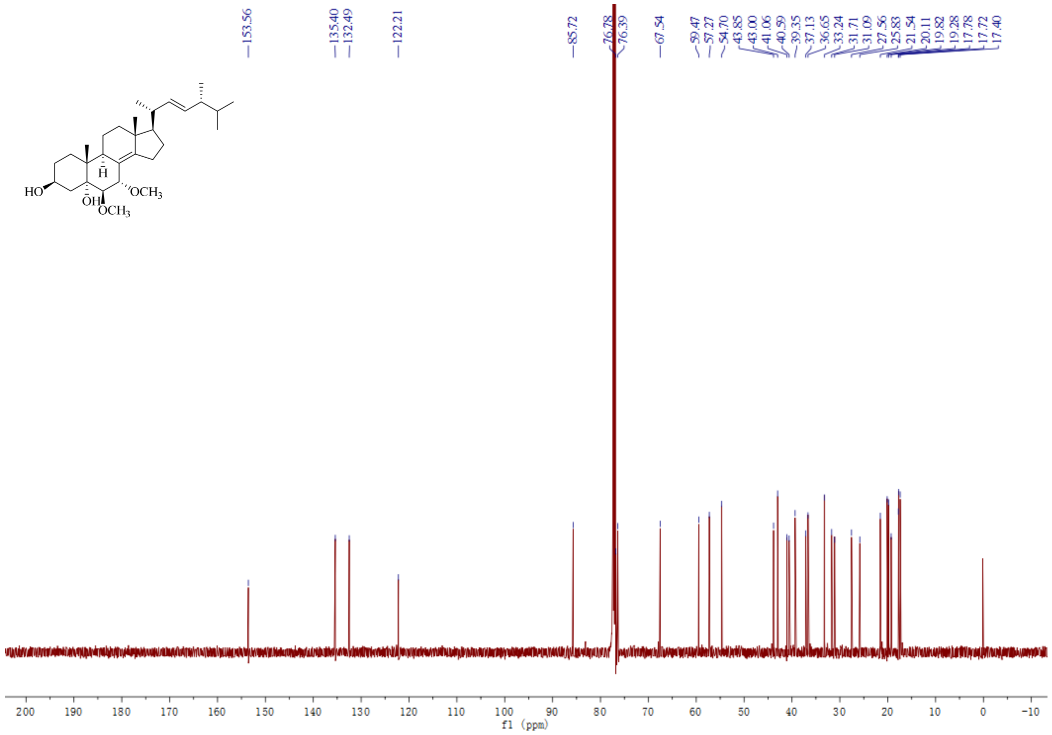

Figure S4. ^{13}C NMR spectrum (150 MHz, $CDCl_3$) of compound **1**

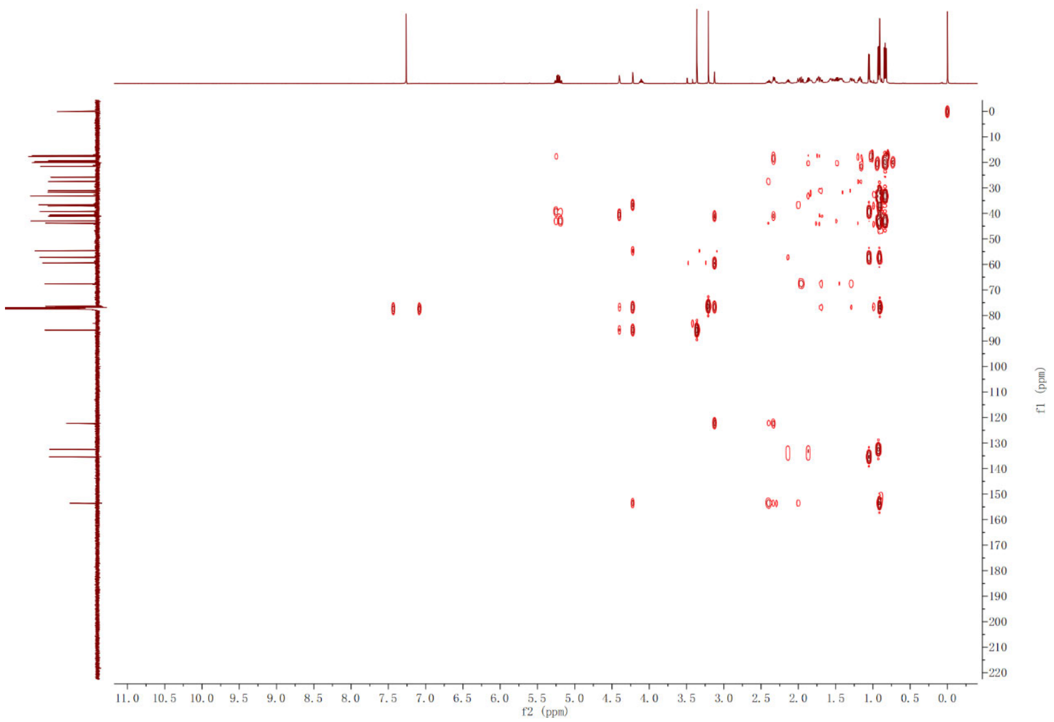

Figure S5. HMBC spectrum (600 MHz, $CDCl_3$) of compound **1**

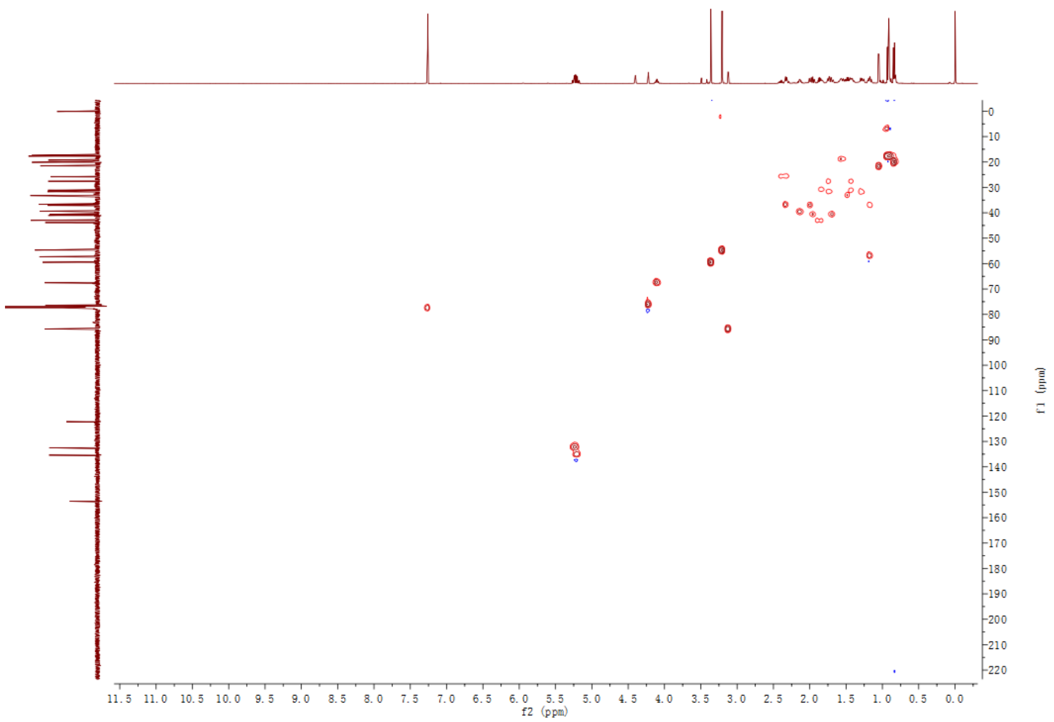

Figure S6. HSQC spectrum (600 MHz, $CDCl_3$) of compound **1**

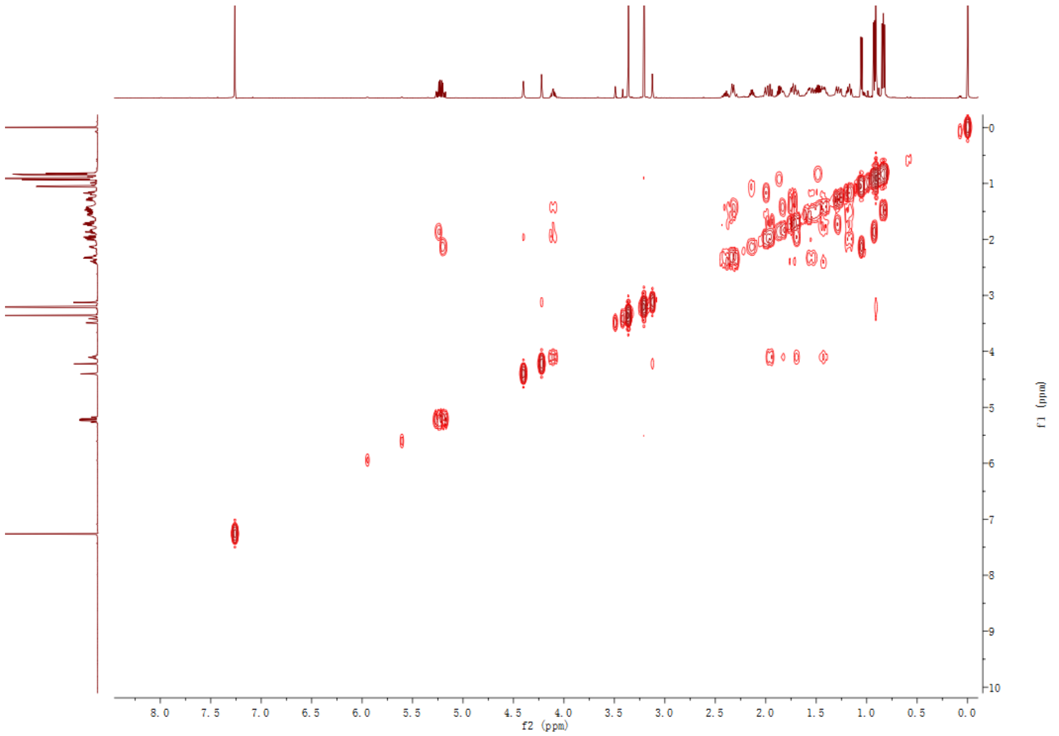

Figure S7. ^{1}H-^{1}H COSY spectrum (600 MHz, $CDCl_3$) of compound **1**

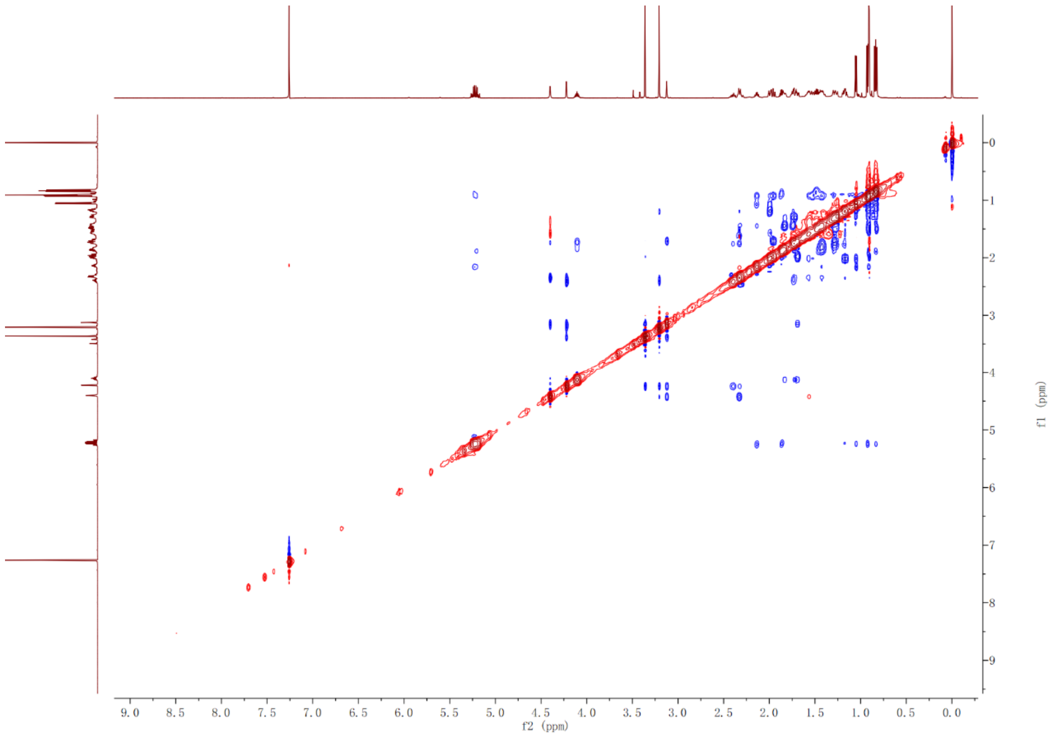

Figure S8. NOESY spectrum (600 MHz, $CDCl_3$) of compound **1**

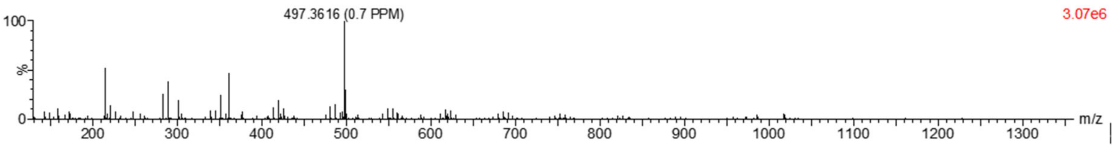

Figure S9. HRESIMS spectrum of compound **1**

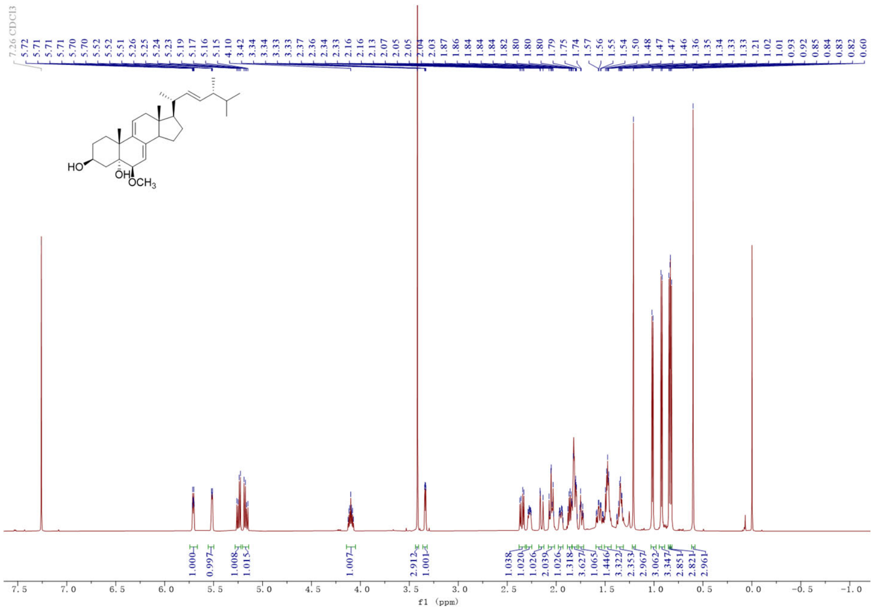

Figure S10. ^{1}H NMR spectrum (600 MHz, $CDCl_3$) of compound **2**

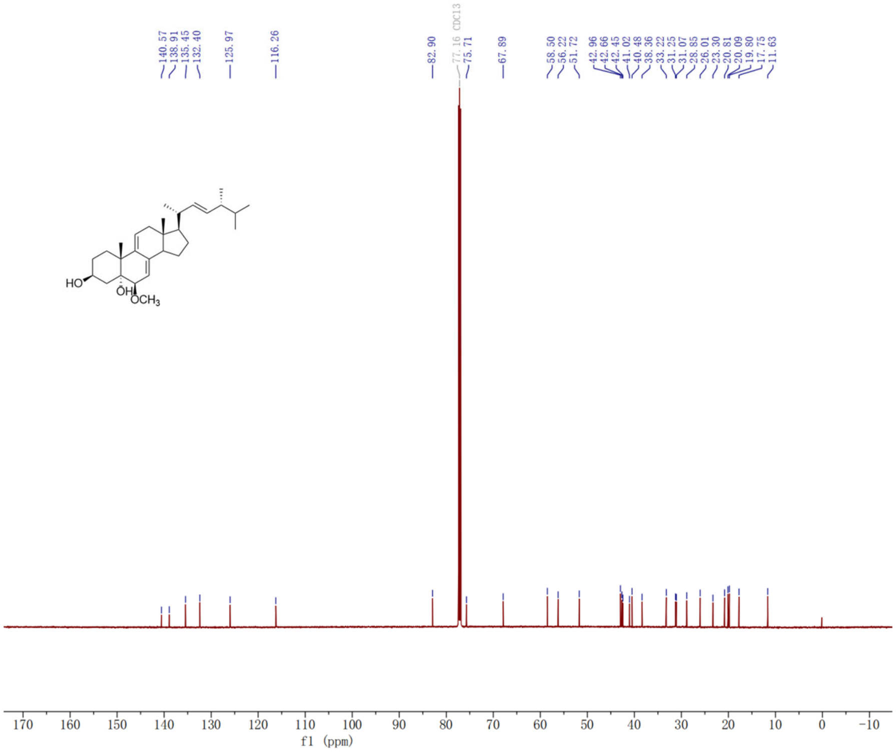

Figure S11. ^{13}C NMR spectrum (150 MHz, $CDCl_3$) of compound **2**

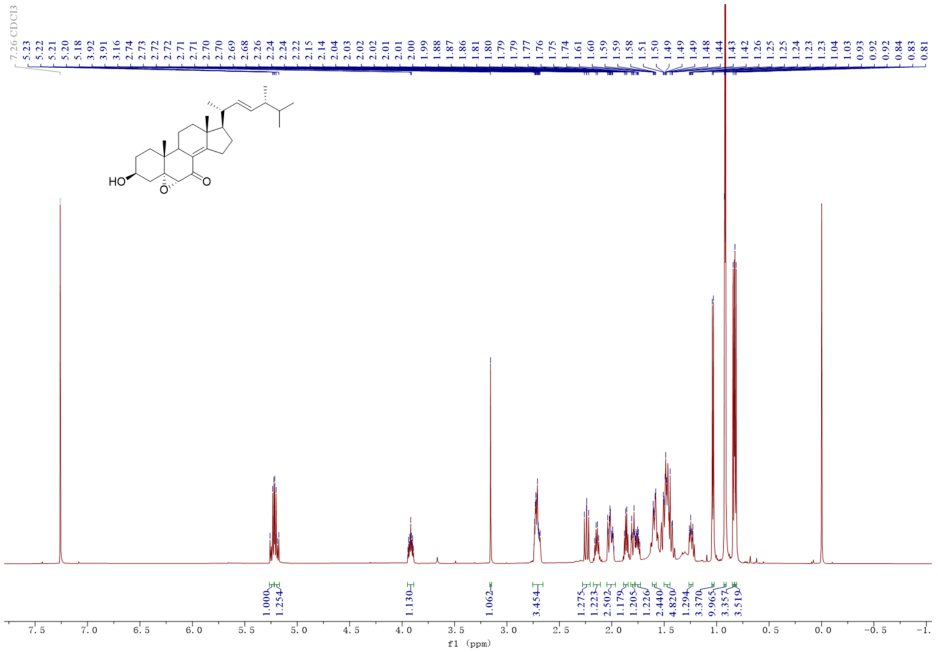

Figure S12. ^{1}H NMR spectrum (600 MHz, $CDCl_3$) of compound **3**

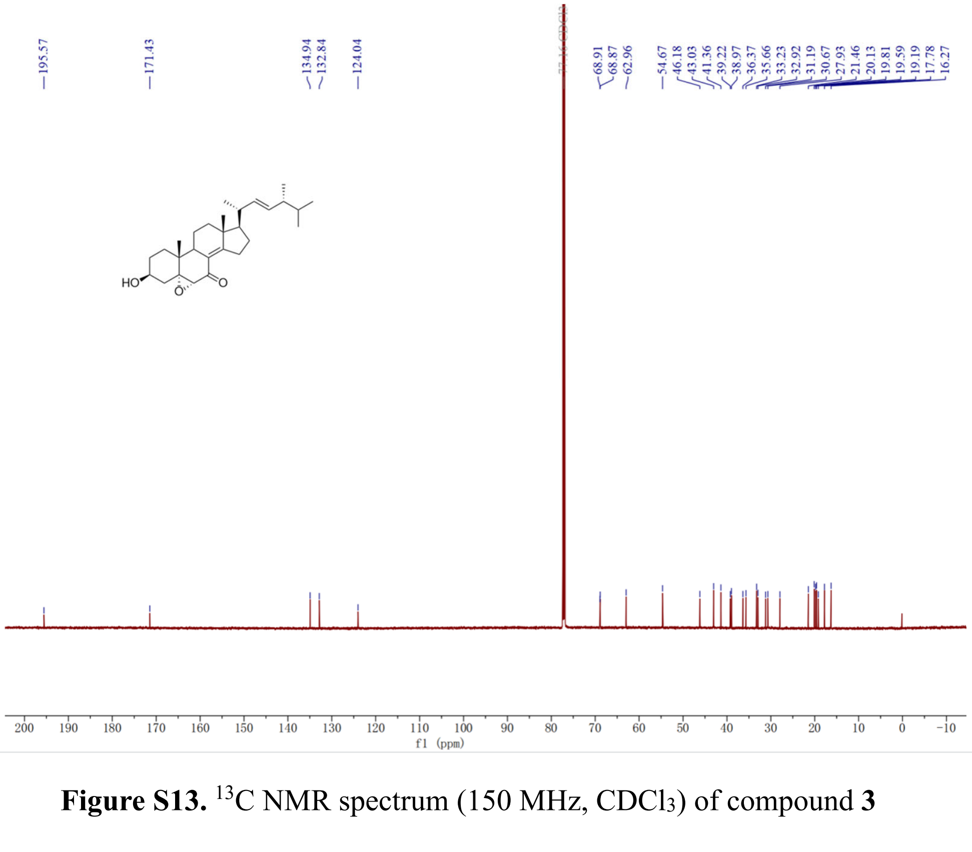

Figure S13. ^{13}C NMR spectrum (150 MHz, $CDCl_3$) of compound **3**

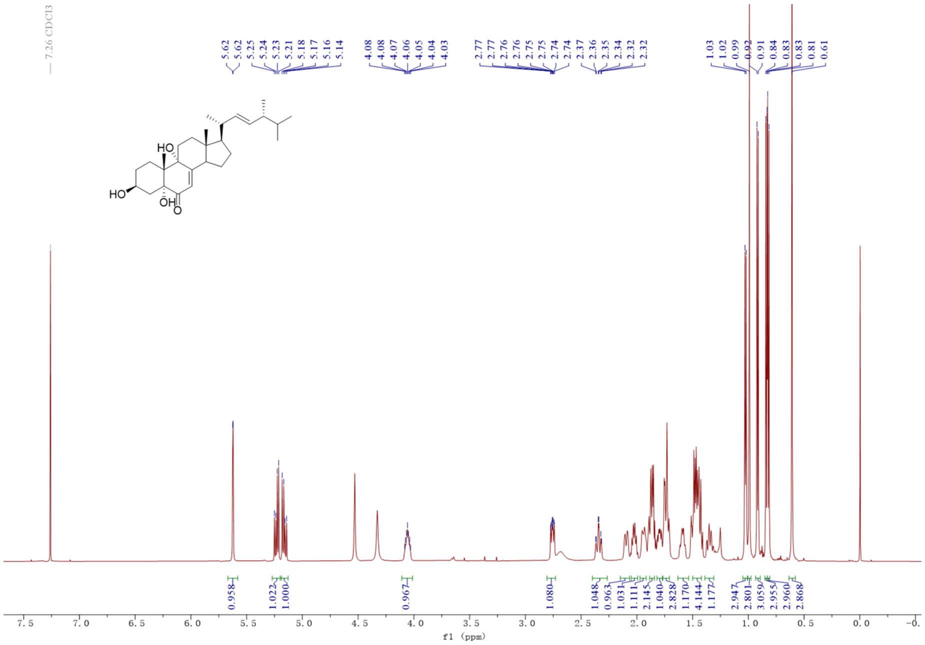

Figure S14. ^{1}H NMR spectrum (600 MHz, $CDCl_3$) of compound **4**

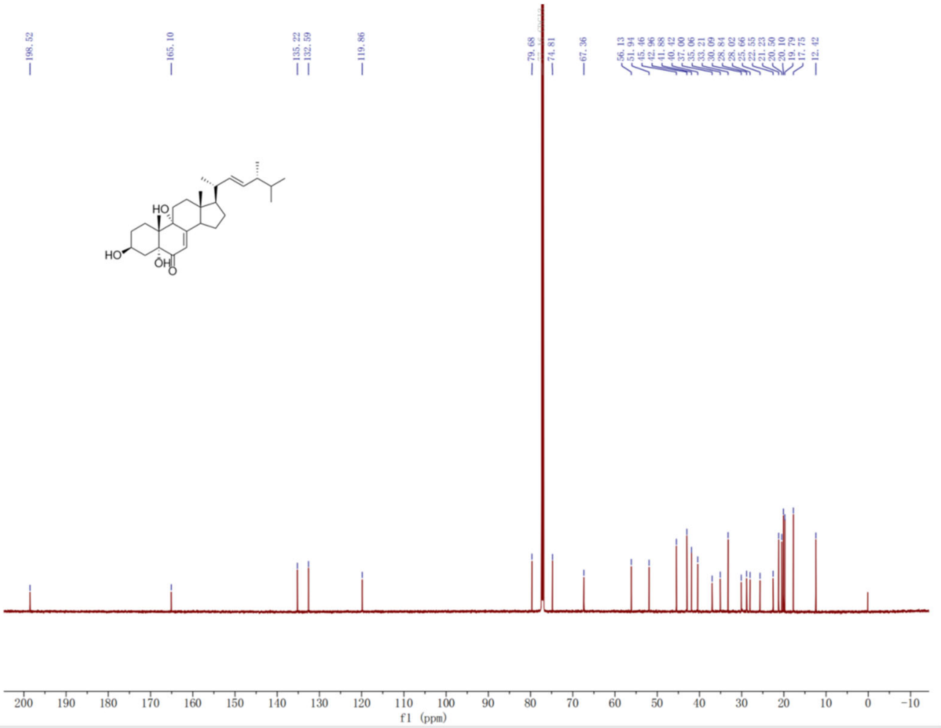

Figure S15. ^{13}C NMR spectrum (150 MHz, $CDCl_3$) of compound **4**

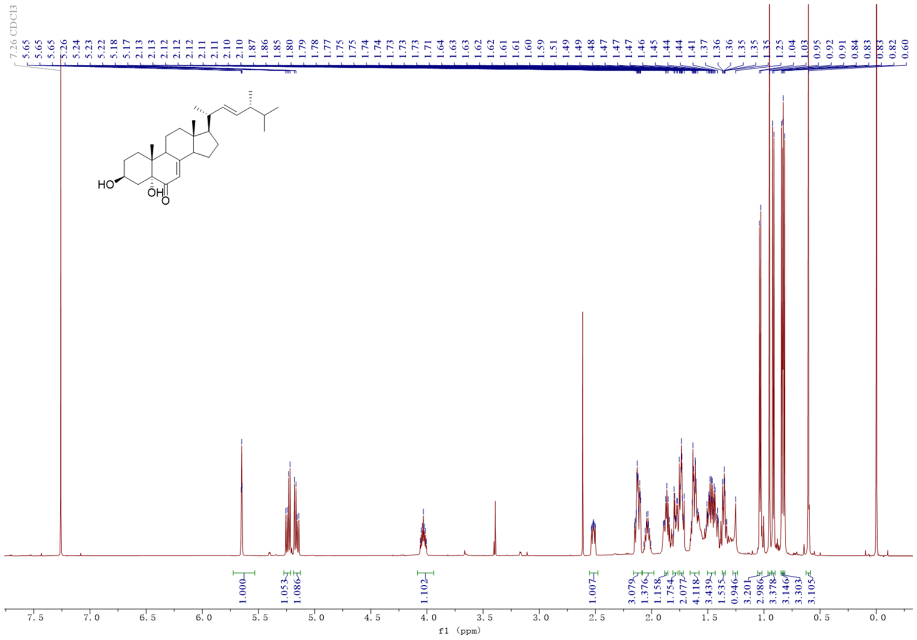

Figure S16. ^{1}H NMR spectrum (600 MHz, $CDCl_3$) of compound **5**

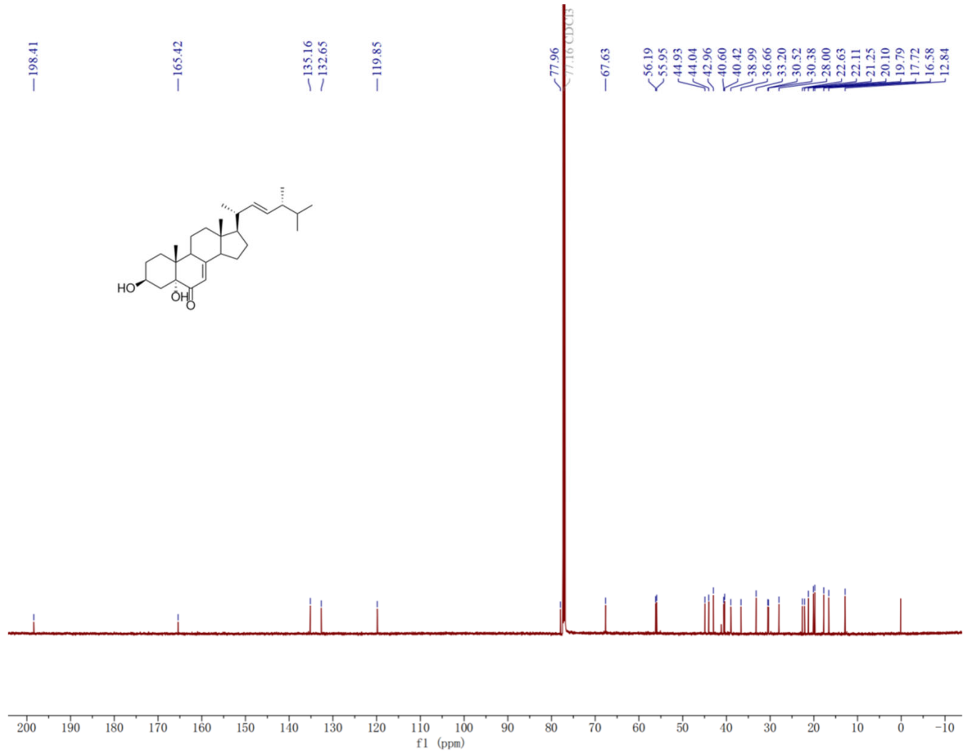

Figure S17. ^{13}C NMR spectrum (150 MHz, $CDCl_3$) of compound **5**

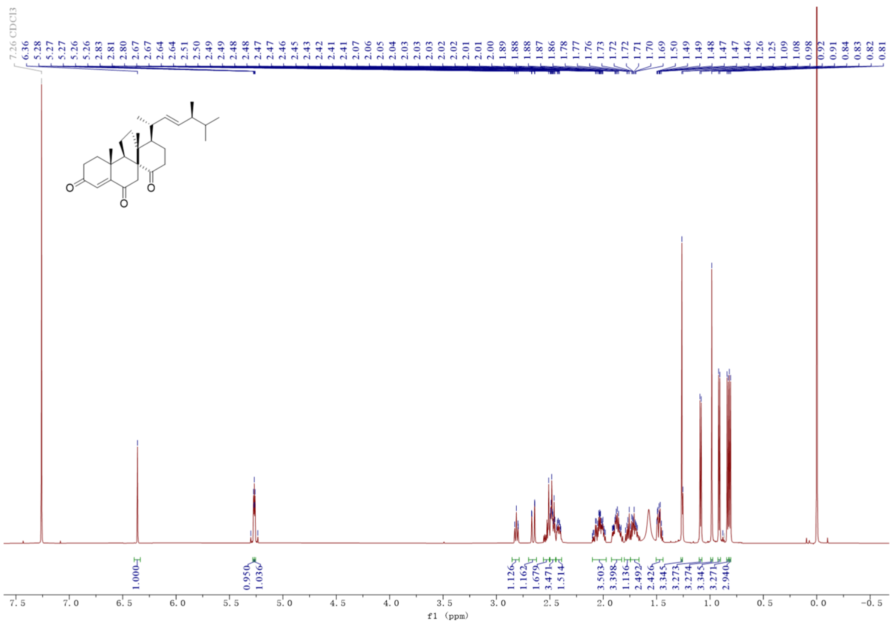

Figure S18. ^{1}H NMR spectrum (600 MHz, $CDCl_3$) of compound **6**

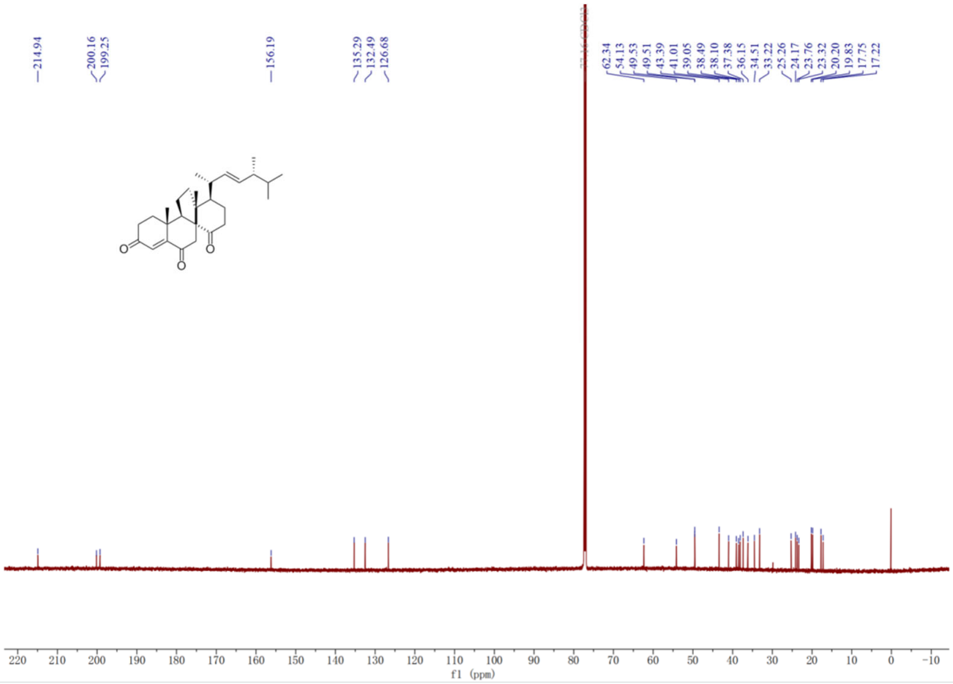

Figure S19. ^{13}C NMR spectrum (150 MHz, $CDCl_3$) of compound **6**

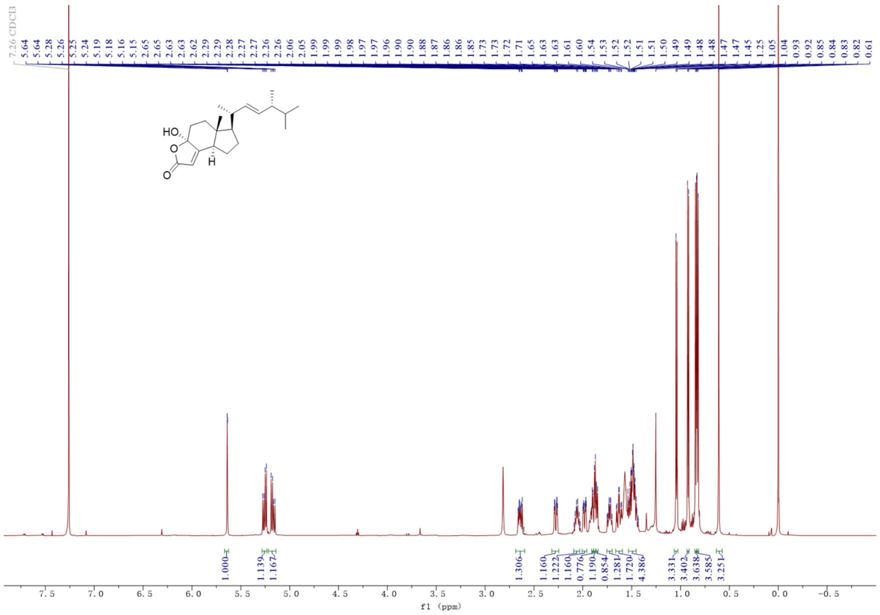

Figure S20. ^{1}H NMR spectrum (600 MHz, $CDCl_3$) of compound **7**

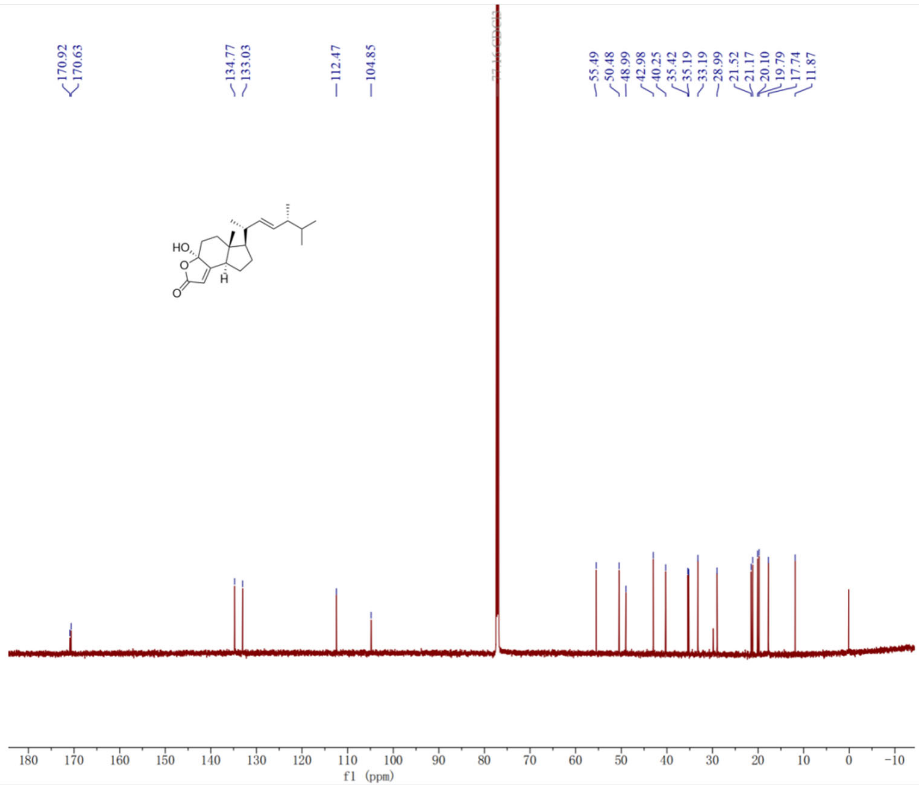

Figure S21. ^{13}C NMR spectrum (150 MHz, $CDCl_3$) of compound **7**

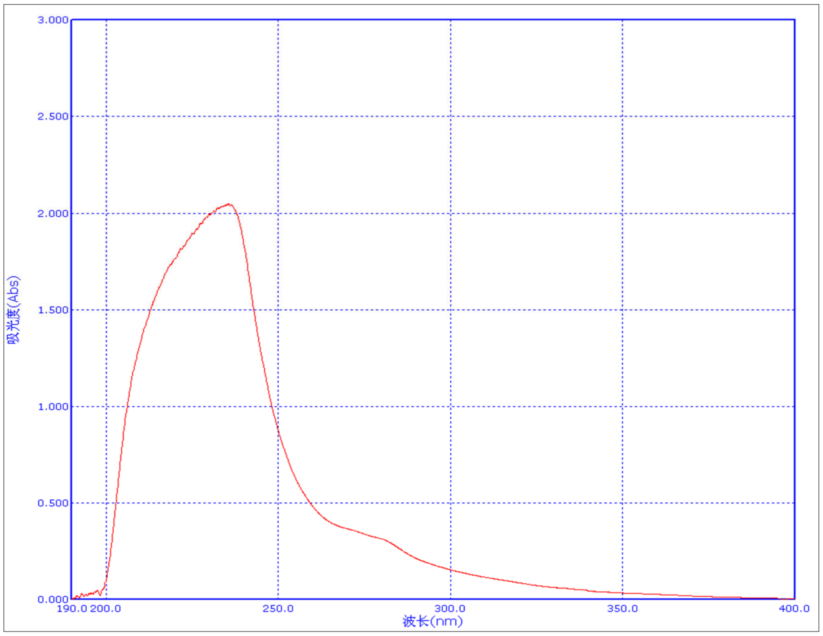

Figure S22. UV spectrum of compound **8**

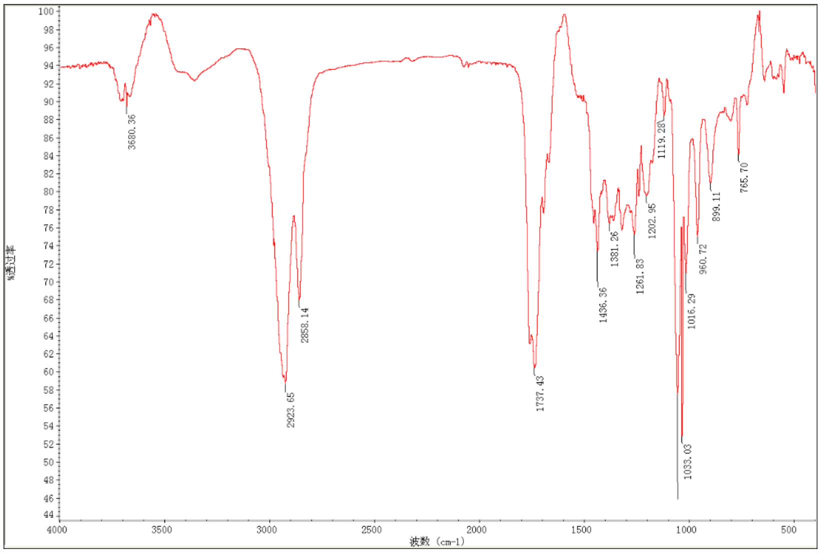

Figure S23. IR spectrum of compound **8**

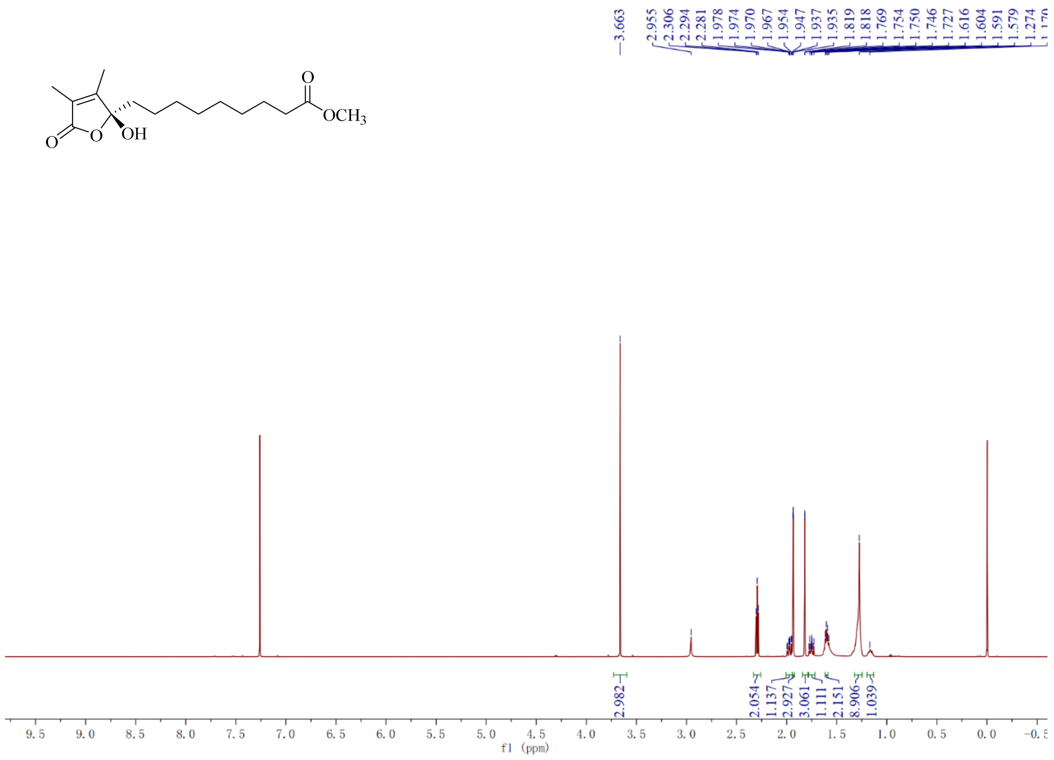

Figure S24. ^{1}H NMR spectrum (600 MHz, $CDCl_3$) of compound **8**

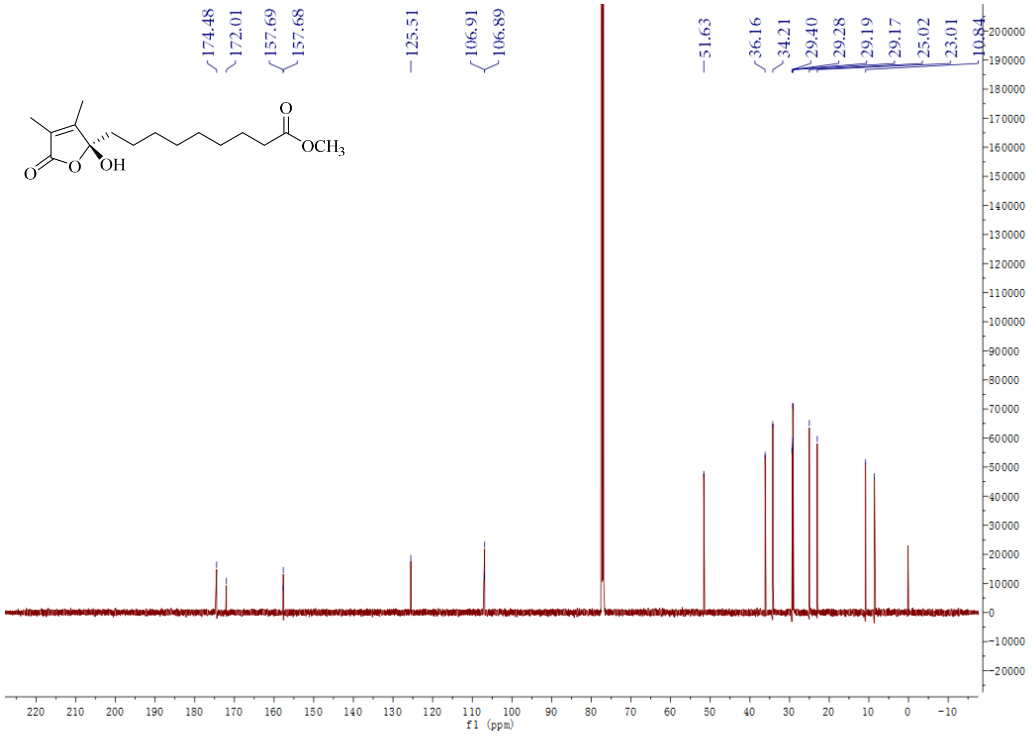

Figure S25. ^{13}C NMR spectrum (150 MHz, $CDCl_3$) of compound **8**

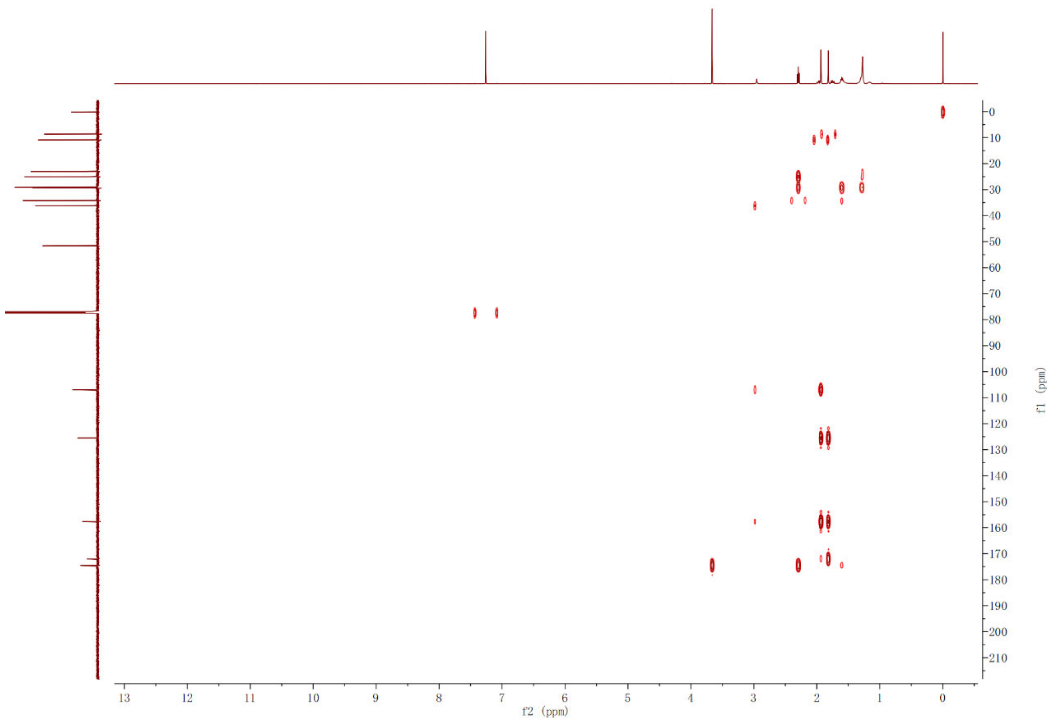

Figure S26. HMBC spectrum (600 MHz, $CDCl_3$) of compound **8**

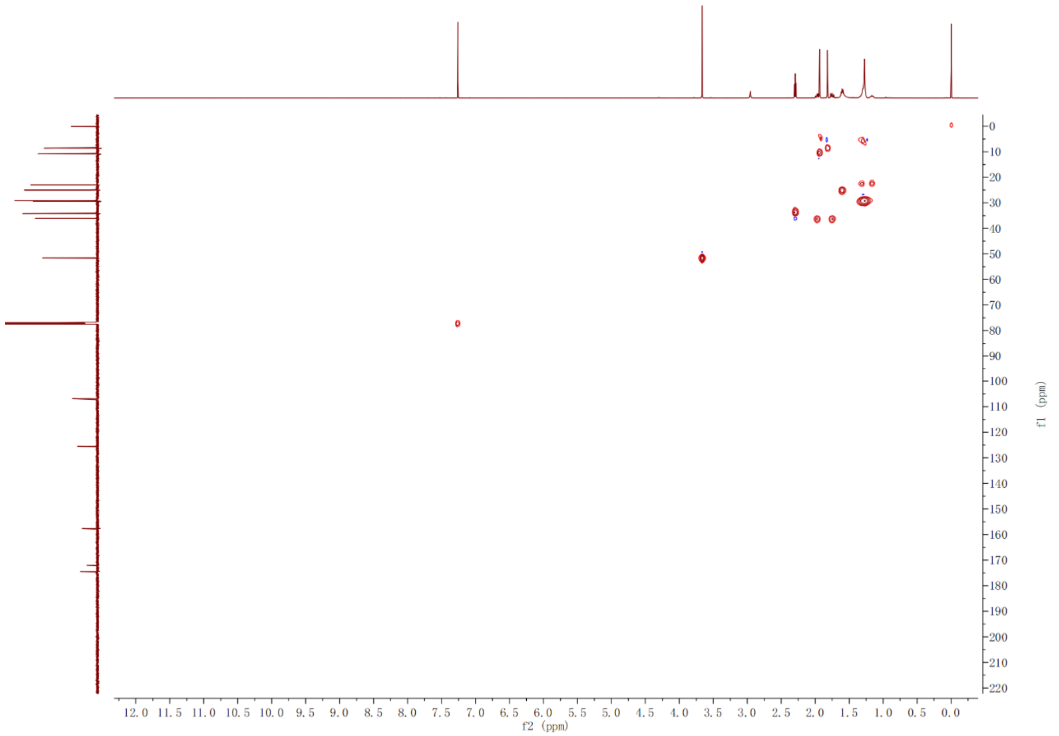

Figure S27. HSQC spectrum (600 MHz, $CDCl_3$) of compound **8**

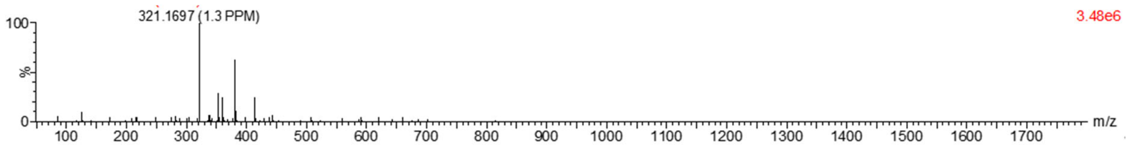

Figure S28. HRESIMS spectrum of compound **8**

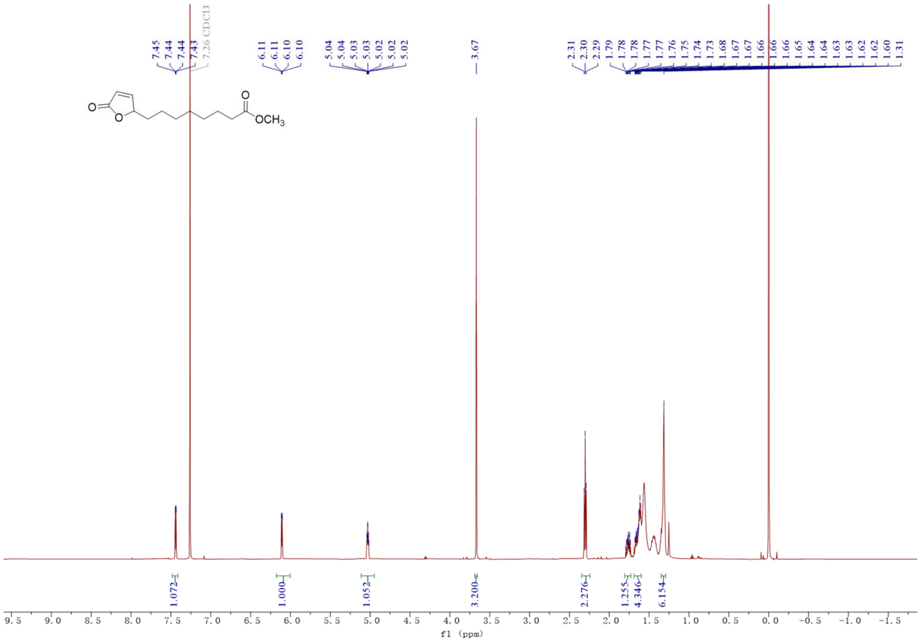

Figure S29. ^{1}H NMR spectrum (600 MHz, $CDCl_3$) of compound **9**

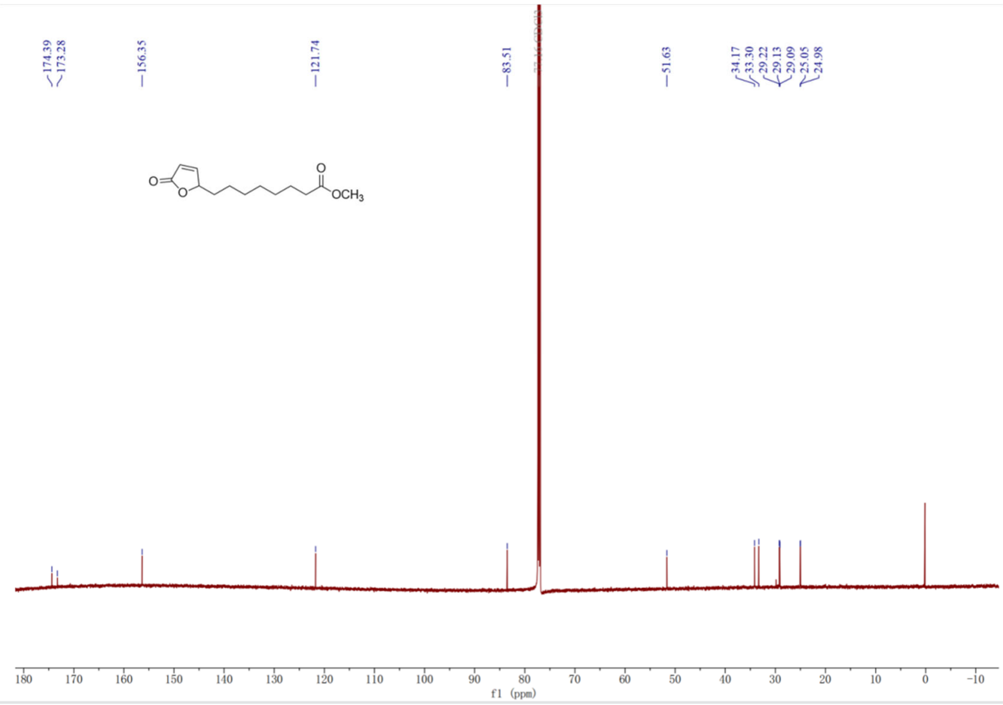

Figure S30. ^{13}C NMR spectrum (150 MHz, $CDCl_3$) of compound **9**

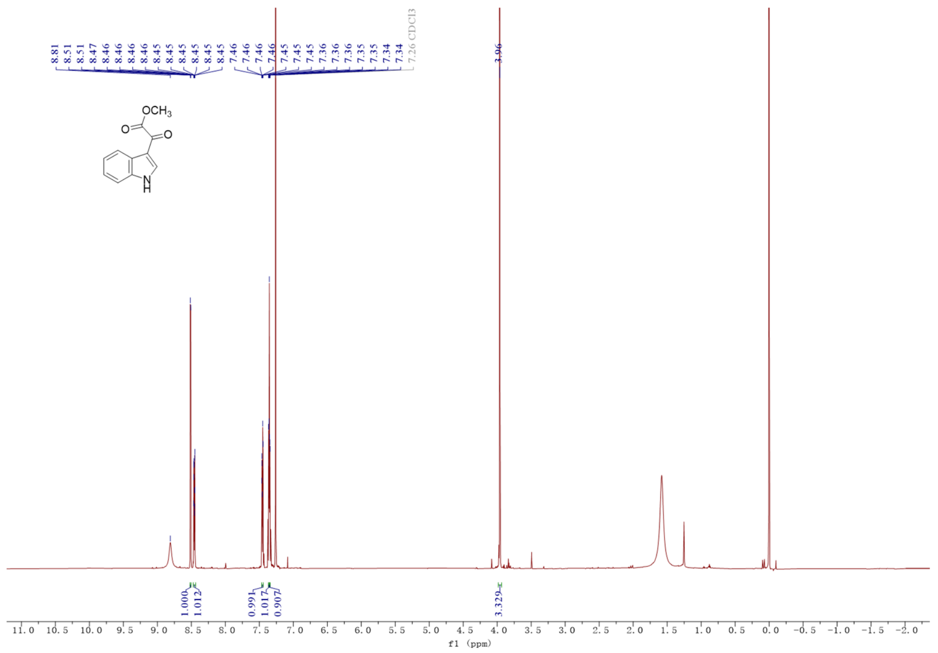

Figure S31. ^{1}H NMR spectrum (600 MHz, $CDCl_3$) of compound **10**

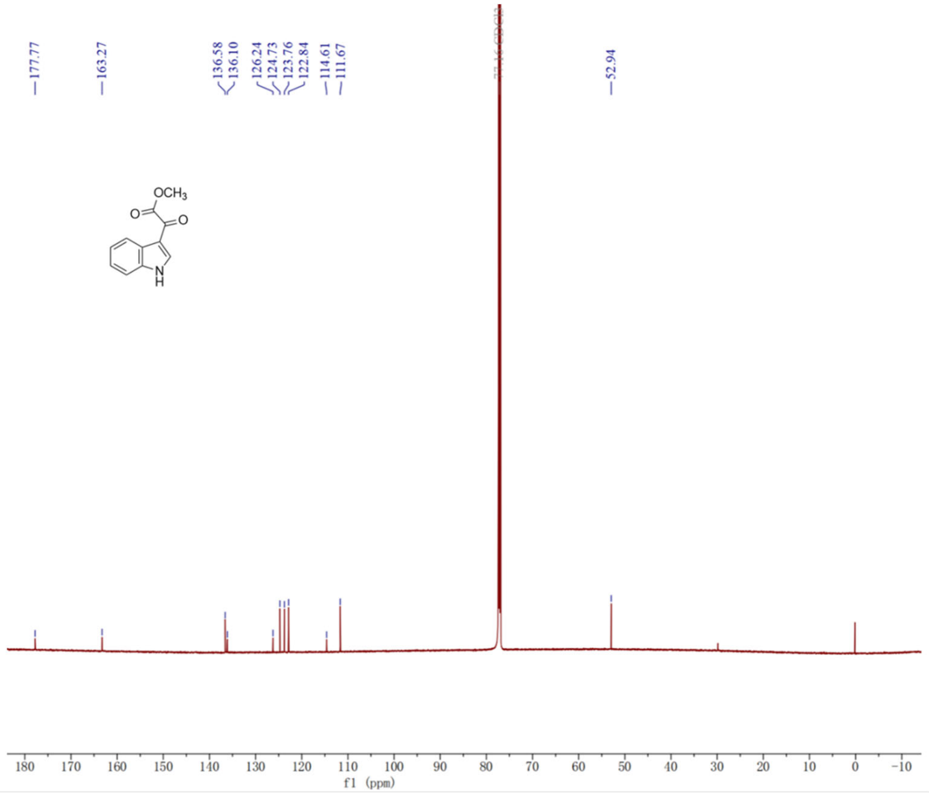

Figure S32. ^{13}C NMR spectrum (150 MHz, $CDCl_3$) of compound **10**

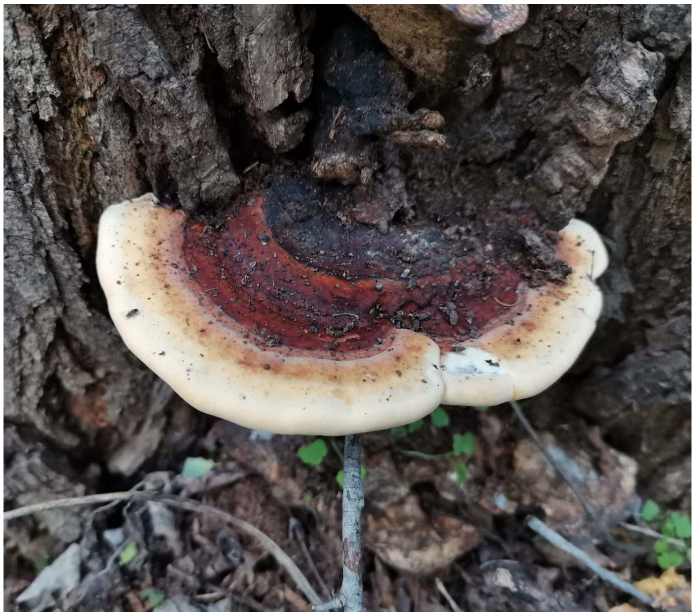

Figure S33. Basidiomes of *Vanderbylia robiniophila*. Photo taken by Hai-Sheng Yuan in China, 2020.

The rDNA-ITS sequence of the experimental strain IFP1101:

ACCTGCGGAAGGATCATTATCGAGTTTTGAAAGGGGTTGTAGCTGGCC
TTCCGAGGCATGTGCACGCCCCGCTCAATCCACTCTACACCTGTGCACTTA
CTGTGGGTTTCGGAGGTGAAGCGTGCTTTCGCTCGCGGATCTAACGGGCCC
GCGTTTTACTACAAACACTTTAAAGTAAACGAACGTGTATCGCGATGTAAC
GCATCTATATACAACTTTCAGCAACGGATCTCTTGGCTCTCGCATCGATGAA
GAACGCAGCGAAATGCGATAAGTAATGTGAATTGCAGAATTCAGTGAATCA
TCGAATCTTTGAACGCACCTTGCGCTCCTTGGTATTCCGAGGAGCATGCCT
GTTTGAGTGTCATGAAATTCTCAACCTACCGGTCTTTGCGGATCGGTAAGG
CTTGGACTTGGAGGCTTGTCGGCCCGTGTGGTCGACTCCTCTCAAATGCAT
TAGCCTGGTTCCTTGCGGATCGGCTCTCGGTGTGATAATTGTCTACGCCGCG
ACCGTGAAGCGTTTGGCTGGCTTCTAACCGTCTCGATGGAGACAACTTTCT
GACCTCTGACCTCAAATCAGGTAG
